# Trace metal accumulation through the environment and wildlife at two derelict lead mines in Wales

**DOI:** 10.1016/j.heliyon.2024.e34265

**Published:** 2024-07-09

**Authors:** Andrea Sartorius, Matthew F. Johnson, Scott Young, Malcolm Bennett, Kerstin Baiker, Paul Edwards, Lisa Yon

**Affiliations:** a School of Veterinary Medicine and Science, University of Nottingham, Sutton Bonington, UK; b School of Geography, University of Nottingham, Nottingham, UK; c School of Biosciences, University of Nottingham, Sutton Bonington, UK; d Natural Resources Wales, Cardiff, UK

**Keywords:** Legacy pollutants, Mining, Trace metals, Wildlife health

## Abstract

Trace metal pollution is globally widespread, largely resulting from human activities. Due to the persistence and high toxicity of trace metals, these pollutants can have serious effects across ecosystems. However, few studies have directly assessed the presence and impact of trace metal pollution across ecosystems, specifically across multiple environmental sources and animal taxa. This study was designed to assess the environmental health impacts of trace metal pollution by assessing its extent and possible transfer into wildlife in the areas surrounding two abandoned metalliferous mine complexes in Wales in the UK. Water, sediment, and soil at the mine sites and in areas downstream had notably elevated concentrations of Pb, Zn, and, to a lesser extent, Cd and Cu, when compared to nearby control sites. These high trace metal concentrations were mirrored in the body burdens of aquatic invertebrates collected in the contaminated streams both at, and downstream of, the mines. Wood mice collected in contaminated areas appeared to be able to regulate their Zn and Cu tissue concentrations, but, when compared to wood mice from a nearby control site, they had significantly elevated concentrations of Cd and, particularly, Pb, detected in their kidney, liver, and bone samples. The Pb concentrations found in these tissues correlated strongly with local soil concentrations (kidney: ρ = 0.690; liver: ρ = 0.668, bone: ρ = 0.649), and were potentially indicative of Pb toxicity in between 10 % and 82 % of the rodents sampled at the mine sites and in areas downstream. The high trace metal concentrations found in the environment and in common prey species (invertebrates and rodents) indicates that trace metal pollution can have far-reaching, ecosystem-wide health impacts long after the polluting activity has ceased, and far beyond the originating site of the pollution.

## Highlights

• Ecosystem trace metal pollution was assessed in and around two derelict metal mines.
• High environmental trace metal concentrations were found in and around mine sites.
• Local aquatic invertebrates had notably elevated trace metal body burdens.
• Exposed rodents had lead concentrations which could be indicative of toxicity.
• Trace metals can have far-reaching impacts beyond the original site of pollution.

## Graphical abstract

**Figure:**
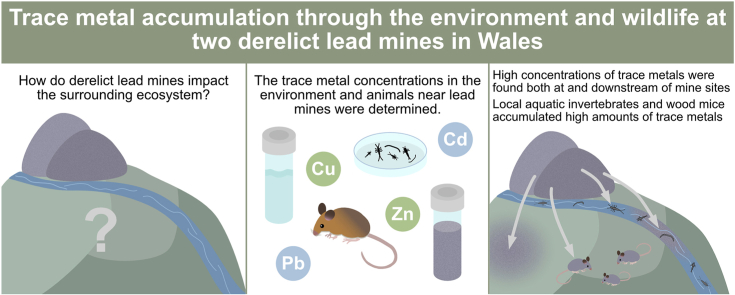

## 1 Introduction

Potentially toxic metals have been mined globally for thousands of years [1,2]. An unintended side effect of these efforts is the production of large amounts of mining waste contaminated with high concentrations of trace metals, elements that are normally found in the Earth's crust at low concentrations [3,4]. This waste can pose ecosystem-wide health risks through the exposure of wildlife and human populations to elements that are toxic at relatively low concentrations [4].

During mining activities, environmental trace metal contamination commonly occurs through aerial transmission of mining dust or the release of contaminated wastewater [5,6]. Once processed, mining waste materials are frequently deposited in ‘spoil heaps’, which are typically located at the mine site. These spoil heaps are large, unstable mounds of material with high trace metal concentrations, and are rarely remediated, even after mine closures [3]. Wind and water erosion of spoil heaps is common, especially since, with vegetative growth inhibited by metal toxicity, there are no plant roots to help stabilise the structure of these heaps [7]. The redistribution of metals may further increase in the future, as flooding and extreme weather events become more common due to climate change [8,9]. As trace metals do not degrade, high concentrations of trace metals can continue to contaminate surrounding environments even long after mining activities have ceased [10,11].

Animals living in metal-contaminated areas are routinely exposed to these potentially toxic metals, primarily through drinking water, diet, or inadvertent soil ingestion [[12], [13], [14], [15]]. There is ample evidence that animals living in trace metal contaminated sites generally have elevated trace metal body burdens [16]. However, few field studies have directly investigated trace metal concentrations across the environment and multiple resident animal taxa to understand the possible ecosystem-wide effects of metal contamination, particularly in terrestrial, polluted sites [12,13]. Therefore, the aims of the current study were [1] to determine the extent of trace metal contamination at and downstream of two derelict metalliferous mines in Wales, and [2] to assess the transfer of trace metals into resident wildlife at low trophic levels, specifically into aquatic invertebrates and rodents. The investigation focused on two ‘non-essential’ metals (metals that serve no metabolic function), Pb and Cd, and two ‘essential’ metals (metals that are necessary for life in small concentrations), Cu and Zn, to examine any differences in the distribution and accumulation of these two classes of trace metals in metal-polluted environments [4,17].

## 2 Methods

### 2.1 Sites

**Fig. 1:**
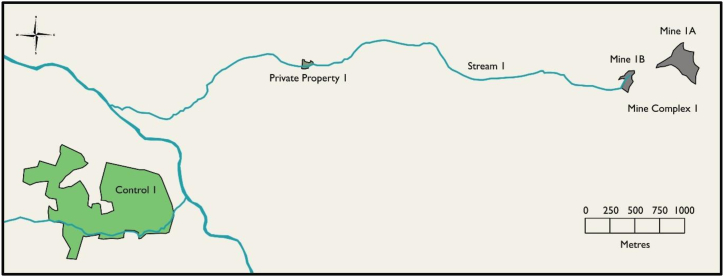
Map of Study Area 1. Grey represents the mine sites, grey-green represents the private property, green represents the control site, and blue represents waterways.

**Fig. 2:**
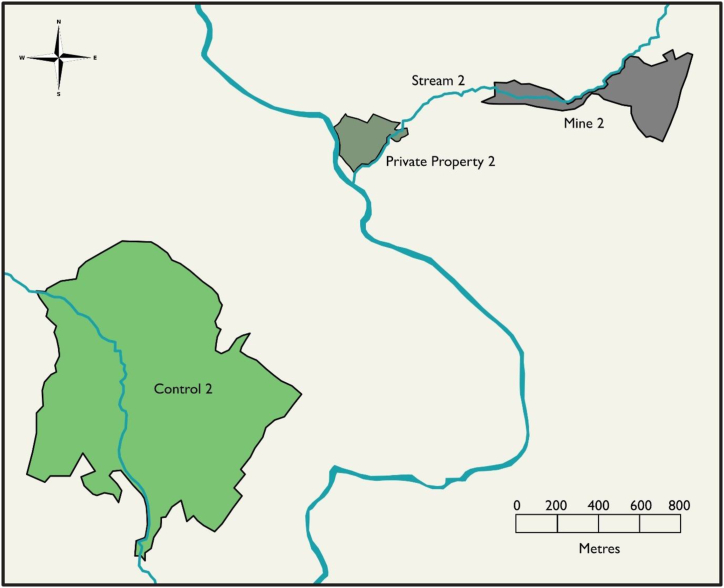
**Map of Study Area 2.** Grey represents the mine sites, grey-green represents the private property, green represents the control site, and blue represents waterways.

Sampling efforts were focused on two derelict metalliferous Pb mine sites and the surrounding areas in mid-Wales in the UK. Samples were collected in May and October 2019 and in September 2021 from two mine sites, two private properties downstream of the mines, and two control sites (one near each mine) (Fig. 1, Fig. 2). To preserve anonymity, the sampled areas are referred to as Areas 1 and 2, with the mine, stream, private property, and control sites in each area given the corresponding area number. Area 1 contained: (i) Mine Complex 1 (made up of Mine 1a and Mine 1b), (ii) Stream 1, which flowed through Mine 1b and Private Property 1, (iii) Private Property 1, which was approximately 4 km downstream of Mine 1b, and (iv) Control 1, which was approximately 6 km from Mine 1b (Fig. 1). Area 2 contained: (i) Mine 2, (ii) Stream 2, which flowed through Mine 2 and Private Property 2, (iii) Private Property 2, which was approximately 1 km downstream of Mine 2, and (iv) Control 2, which was approximately 3 km from Mine 2 (Fig. 2).

Mine Complex 1 and Mine 2 were primarily Pb mines, though Cu was also infrequently extracted at Mine 2. These mines had both been closed for around a hundred years prior to this study. Remediation works, focusing on clay-capping some of the spoil heaps and diverting water from flowing through mine waste, were undertaken at Mine 1a between 2011 and 2018, but these did not completely remove the trace metal pollution at this site (Natural Resources Wales, pers. comm.). Streams 1 and 2 flowed directly through their respective mine sites (passing by large spoil heaps) and the respective downstream private properties before joining larger rivers. The private properties sampled during this study were both small working farms. The control sites were both woodland parks, primarily forested and minimally managed, and contained small streams similar in size to those found at the mine sites. Before being selected as control sites, water, sediment, and soil samples were collected from these sites and tested to confirm that there was minimal trace metal contamination.

### 2.2 Sample collection

Water samples were collected across all sites by rinsing a 20 mL plastic Discardi II syringe (BD, Franklin Lakes, New Jersey, United States) with water at the stream, then filtering an 18 mL aliquot of the water through a polyethersulfone 0.22 μm filter (Chromatography Direct, Runcorn, United Kingdom) and into a 20 mL plastic universal tube. Four samples were collected from each site, two of which were stored in otherwise empty tubes, and two of which were stored in tubes containing 2 mL 10 % Primar grade HNO_3_.

Sediment samples were collected at all water collection sites. Sediment was manually scooped from the riverbed substrate, targeting patches of visibly fine sediment (<5 mm). Sediment was stored in a 50 mL conical centrifuge tube, and any excess water was poured off at the time of collection.

Soil samples were collected along rodent trapping transects (described below). Sub-samples were collected at each trap site along the transect, as well as approximately 5 m to the left and right of the traps (when walking the transect). All sub-samples were deposited within the same bag and mixed thoroughly.

Aquatic invertebrates were sampled in streams at all sites following the Environment Agency's standard protocol [18,19] consisting of a 3 min ‘kick sample’, plus a 1 min hand search, using a 1 mm^2^ mesh net with an opening of 0.25 m wide and 0.22 m deep. The operator moved systematically upstream, ensuring that different habitats were proportionally sampled. The collected invertebrate communities were stored in alcohol. In total, 1794 invertebrate individuals were collected; 787 across six points at the mine sites, 376 across 3 points at the private properties, and 631 across three points at the control sites.

Rodents were collected in May and October 2019 and in September 2021. In May 2019, rodents were trapped at Private Property 1, Private Property 2, and Mine Complex 1; in October 2019, rodents were trapped at all six sites; in September 2021, rodents were trapped at Mine Complex 1, Mine 2, and Control 2. Rodents were trapped using Longworth small mammal traps with shrew holes (Penlon Ltd., Oxford, UK) set along transects in each site. The transects were approximately 20 m long, with two traps set every 5 m. At Private Properties 1 and 2, Stream 1 and Stream 2, respectively, were suspected to be the primary sources of the trace metal contamination from the mine sites, so trapping transects were preferentially placed along these stream banks. For consistency, transects at the mine and control sites were also primarily situated along streams whenever possible. Transects located away from streams were placed in areas that were identified as containing suitable habitat for rodents, such as field boundaries ([20], M. Bennett, pers. comm.). As the traps were strategically placed in order to maximize capture rates, no population estimates can be made from the trapping frequency data.

Seven transects (six at Property 1) were set at each sampled site in May 2019, and five transects were set at each sampled site in October 2019 and September 2021. The number of transects were increased in spring to balance the expected lower rodent populations in spring when compared to autumn [21,22]. Each Longworth trap was prepared with rodent bedding and was baited with bird seed and a small portion of a fresh vegetable for moisture and nutrition (cucumber or carrot). All traps were checked in both the morning and evening to minimise the time any captured rodent spent in the trap. When checking the traps, all closed traps were collected and replaced with fresh traps in the same location. If a closed trap contained a rodent, the species, sex, and age group (juvenile or adult) were recorded. The animal was euthanised using cervical dislocation, with death confirmed by exsanguination, and the kidney, liver, and femur were removed and stored at −20 °C.

### 2.3 Sample processing

Trace metal concentrations were determined in the filtered, acidified water samples using an Inductively Coupled Plasma Mass Spectrometer (ICP-MS) (Model ICAP-Q; Thermo Fisher Scientific, Bremen, Germany). Dissolved organic carbon (DOC) was determined in the filtered, non-acidified water samples using a TOC-VCPH instrument (Shimadzu Corporation, Kyoto, Japan).

The sediment and soil samples were air-dried, sieved to <2 mm with a stainless steel sieve, and ground into fine powder using a Retsch PM 400 planetary ball mill (Retsch, Haan, Germany). Duplicates of 0.4 g of each sample were digested in Aqua Regia (1 mL HNO_3_ and 3 mL HCl) at 95 °C for 2 h using a teflon-coated graphite hotplate block digester (Analab, Bischeim, France). The samples were then cooled and dispensed into volumetric flasks; the volume was then made up to 50 mL with MilliQ water (18.2 MΩ cm; Millipore Corporation, Darmstadt, Germany). The solutions were then further diluted 1:10 with MilliQ water prior to elemental analysis by ICP-MS. A certified soil reference material (NIST 2711A, Montana soil) was run for quality assurance purposes; recovery values were: Pb (93.1 %), Cd (104.5 %), Zn (99.9 %), and Cu (91.8 %). For each element, the operational limit of detection (LOD) was calculated as three times the standard deviation of the concentrations measured in 10 blank digestion samples run alongside the samples [23]. A value of 0.5*LOD was ascribed to samples where the elemental concentration was lower than the LOD [24].

The Unified BARGE (the BioAccessibility Research Group of Europe) Method (UBM) was used to determine the bioaccessibility of trace metals in the soil samples collected along the rodent transects [25,26]. The rodent transect soil samples were run in triplicate, using 0.47 g of the sieved soil each time. All solution volumes in the original method were scaled down to match the amount of soil used; otherwise, no alterations were made to the UBM [26]. The amended protocol can be found in Appendix C. The final supernatant was diluted 1-in-10 with 2 % HNO_3_ prior to elemental analysis by ICP-MS. After obtaining the final concentrations, the bioaccessibility percentage was calculated for each soil sample as [concentration of bioaccessible metal (mg kg^−1^)/concentration of total metal in sample (mg kg^−1^)] * 100, following BARGE & INERIS [26].

The invertebrates were sorted in the laboratory, with each individual identified to family level, with the exception of Oligochaeta, which were identified to sub-class level. After identification, the invertebrates were freeze dried to a constant mass and prepared for acid digestion. Any invertebrate weighing over 0.01 g (dry weight) was acid digested individually. For invertebrates weighing under 0.01 g, multiple individuals of the same family and collected from the same site and at the same time were pooled together to reach a cumulative weight greater than 0.01 g. In total, 198 invertebrate samples were processed; 75 from the mine sites, 35 from the private properties, and 88 from the control sites. Samples were acid digested and prepared for ICP-MS analysis following a similar protocol to the sediment and soil samples, but with 4 mL 70 % HNO_3_ and 1 mL H_2_O_2_ instead of 1 mL HNO_3_ and 3 mL HCl. A certified reference material for trace elements in biological samples (BRC-185R Bovine Liver [trace elements]) was run for quality assurance purposes; recovery values were: Pb (94.2 %), Cd (103.9 %), Zn (101.2 %), Cu (94.9 %). As with the soil and sediment samples, the LOD was calculated as three times the standard deviation of the concentrations measured in 10 blank digestion samples [23], and any samples where the elemental concentration was lower than the LOD was ascribed the concentration 0.5*LOD [24].

Rodent kidney, liver, and bone samples were freeze dried to a constant mass prior to acid digestion. After freeze drying, bone samples were manually cleaned of soft tissue. Rodent tissues were acid digested through hotplate digestion and prepared for ICP-MS elemental analysis, following the same protocol used for the invertebrate samples. All concentrations were calculated as mg kg^−1^ dry weight; comparisons with published fresh weight concentrations and thresholds were performed assuming 24 % dry matter in kidneys and 26 % dry matter in livers, as reported for wood mice by Ma [27].

### 2.4 Statistical Analysis

Relationships between trace metal concentrations were examined through statistical comparisons in R [28]. Due to a lack of normality in total and bioaccessible soil concentration distributions (determined using Shapiro-Wilk tests), Spearman's correlation coefficients were calculated to determine the possible relationships between the soil trace metal concentrations. The soil concentrations were log-transformed for Supplemental Fig. 1 to highlight possible relationships more readily. Invertebrate metal concentrations were compared across site type and family using Kruskal-Wallis tests (and, when necessary, post-hoc pairwise Wilcox tests), because the concentrations were not normally distributed. Overall rodent metal concentrations were also not normally distributed, so concentrations between rodent species were compared using Kruskal-Wallis tests. However, wood mouse trace metal concentrations were normally distributed when log-transformed, allowing the dataset to fit ANOVA assumptions [29]. The wood mouse log_10_-transformed trace metal concentrations were modelled against the type of site (mine, private property, or control), sex (female or male), age class (juvenile or adult), collection month and year (May 2019, October 2019, September 2021), location (Area 1 or Area 2), and individual site (Mine Complex 1, Mine 2, Private Property 1, Private Property 2, Control 1, Control 2). Tukey post-hoc tests were also run comparing metal concentrations across site types. The correlations between wood mouse tissue and soil trace metal concentrations were determined using Spearman's correlation coefficients, once again chosen due to the non-normal distribution of the soil concentrations. Figures illustrating the statistical tests were generated in R using the ggplot2 package [30].

To decrease the chance of a type I error, Benjamini - Hochberg corrections were utilized for each statistical test, with the total number of sub-tests reflecting the number of factors included in each test type [31]. Adjusted p-values were calculated by multiplying the p-value by the total number of tests, and then dividing by the p-value's rank (when all relevant p-values were ordered smallest to largest), as described in Yekutieli & Benjamini [32].

### 2.5 Threshold comparisons

Water trace metal concentrations were compared to the “Predicted No-Effect Concentrations” (PNECs) defined by the Department for Environment, Food, and Rural Affairs (Defra) [33]. PNECs consider several factors, including dissolved organic carbon (DOC) and calcium concentrations, to estimate trace metal bioavailability and generate threshold trace metal concentrations below which no adverse effects are expected. PNECs are therefore site-specific, even within the same water system. DOC was not measured for every water sample, so, for samples without DOC measurements, DOC values previously measured by Natural Resources Wales in the same locations in the sampled streams were used to generate approximate PNECs (Natural Resources Wales, pers. comm.). PNECs were calculated using the Metal Bioavailability Assessment Tool (M-BAT) Microsoft Excel calculators, generated by the Water Framework Directive United Kingdom Technical Advisory Group [34]. PNECs could only be calculated for Pb, Zn, and Cu, as no PNEC calculator currently exists for Cd.

The sediment sample trace metal concentrations were compared to the Canadian sediment quality guidelines for the protection of aquatic life [35], as no equivalent exists for the UK. These guidelines are composed of two values for each pollutant: the Interim Sediment Quality Guideline (ISQG), above which adverse health effects for aquatic life are possible, and the Probable Effect Level (PEL), above which adverse health effects for aquatic life are probable.

The soil sample trace metal concentrations were compared to the Normal Background Concentrations (NBCs), which were generated by the British Geological Survey (BGS) to indicate ‘normal levels of contaminants’ in different ‘domains’ (areas defined based on soil type and anthropogenic activity) within the UK [36]. In the current study, the NBC values for the ‘Principal’ (background) and ‘Mineralization’ (mining areas) domains resolved for Wales by the BGS in 2013 are referenced for comparison purposes [36]. These can be used to determine if the sampled soil trace metal concentrations were elevated compared to expected soil concentrations in Wales, particularly in mining areas [36]. The soil trace metal concentrations were also compared to the ‘Sludge (Use in Agriculture) Regulations 1989’, which specify the maximum soil trace metal concentrations below which it is permitted to apply sewage sludge (biosolids) to agricultural land [37]. These thresholds were designed to protect against the contamination of agricultural produce, and are broadly similar to previous limits relating to the re-development of contaminated land, so they can provide an approximate indication of contamination levels.

## 3 Results

### 3.1 Environmental samples

**Fig. 3:**
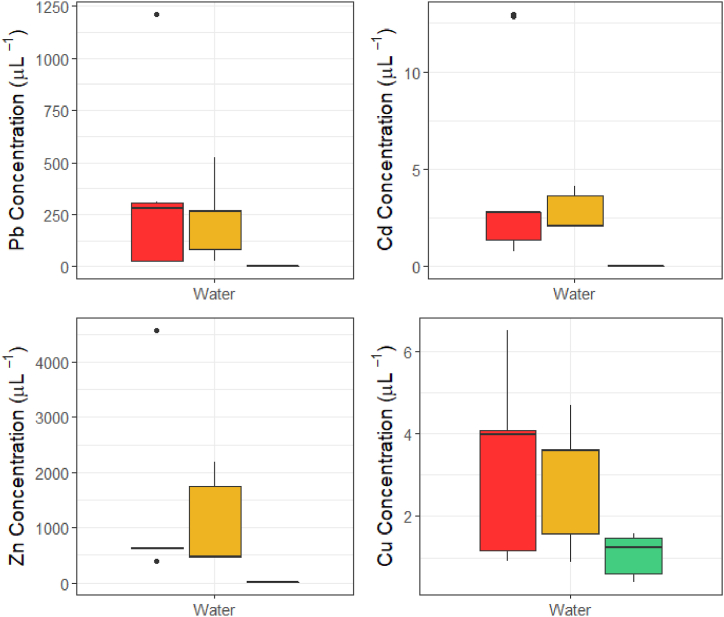
**Water trace metal concentrations across mine, private property, and control sites.** Red represents the mine sites, yellow represents the private properties, and green represents the control sites.

The water samples at the mines and private properties had consistently higher Pb, Cd, Zn, and Cu concentrations than those measured at the control sites (Fig. 3; Table 1). The metal concentrations at the private properties generally resembled those found in the mine sites (Fig. 3). Beyond 4 km in Area 1, and 2 km in Area 2, the streams from the mine sites joined larger waterways contaminated with metals from other sources (not shown in Fig. 1), so determining the full spatial extent of metal contamination in the waterways from the sampled mines was not possible. All sampled sites at the mines and private properties greatly exceeded their site-specific PNECs for Pb and Zn water concentrations (Table 1), indicating that the concentrations of those metals in the streams could cause adverse health effects in resident aquatic organisms.

**Table 1:** **Water trace metal concentrations across the mine, private property, and control sites.** Means are reported as ± standard deviation, ranges are provided in italics. Concentrations are in μg L^−1^. PNECs are reported for Pb, Zn, and Cu; no PNECs can be generated for Cd.

Site	n		Pb	Cd	Zn	Cu
Mine	5	Mean	369 ± 186	4.13 ± 4.69	1370 ± 1690	3.32 ± 2.17
		*Range*	*23.1–1210*	*0.765–13.0*	*384–4570*	*0.905–6.51*
		PNEC^a^^,^^b^	4.93 ± 2.35	–	16.8 ± 5.09	7.81 ± 3.69
Private Property	3	Mean	230 ± 186	2.77 ± 1.07	1040 ± 890	2.89 ± 1.60
		*Range*	*26.5–524*	*2.05–4.15*	*448–2190*	*0.885–4.70*
		PNEC	3.32 ± 0.202	–	12.5 ± 0.754	5.26 ± 0.321
Control	3	Mean	0.360 ± 0.0785	0.0245 ± 0.0170	2.81 ± 0.352	1.06 ± 0.540
		*Range*	*0.280–0.493*	*0.00270–0.0394*	*2.45–3.29*	*0.390–1.58*
		PNEC	7.24 ± 3.70	–	22.2 ± 8.99	11.6 ± 5.91

a Probable No-Effect Concentration.

b 33.

**Fig. 4:**
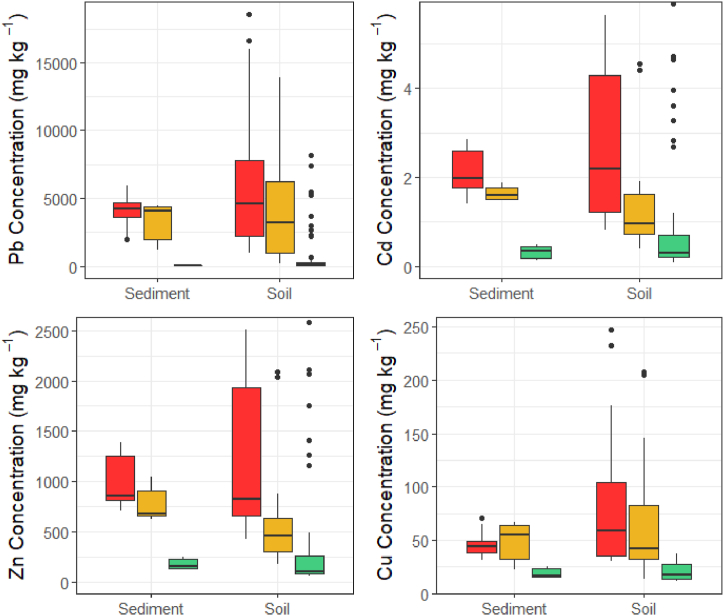
**Sediment and soil trace metal concentrations across mine, private property, and control sites.** Red represents the mine sites, yellow represents the private properties, and green represents the control sites.

Sediment samples collected at the mine and private property sites had consistently higher trace metal concentrations than sediment samples collected at the control sites (Fig. 4; Table 2). All the sediments collected from the mines and private properties exceeded the PEL for Pb and Zn, indicating that adverse health effects in aquatic life were probable, and exceeded the ISQG for Cd, indicating that adverse health effects in aquatic life from Cd exposure were possible (Table 2) [35]. The Cu concentrations also exceeded the ISQG for all mine and private property sampling locations except for two (one at Mine 1b and one at Private Property 1) (Table 2) [35].

**Table 2:** **Sediment trace metal concentrations across the mine, private property, and control sites.** Means are reported as ± standard deviation, ranges are provided in italics. Concentrations are in mg kg^−1^.

Site	n	Pb	Cd	Zn	Cu
Mine	7	4080 ± 1160	4.03 ± 5.17	2030 ± 2850	46.0 ± 11.3
		*1990–5960*	*1.41–16.3*	*703–8820*	*14.7–25.7*
Private Property	3	3270 ± 1590	1.63 ± 0.170	775 ± 187	48.7 ± 19.7
		*1190–4480*	*1.47–1.88*	*619–1050*	*22.8–66.8*
Control	3	64.4 ± 37.3	0.319 ± 0.152	178 ± 55.7	18.8 ± 5.29
		*28.4–126*	*0.134–0.491*	*131–252*	*14.7–25.7*
ISQG^a^^,^^c^		35.0	0.6	123	35.7
PEL^b^^,^^c^		91.3	3.5	315	197

a Interim Sediment Quality Guideline.

b Probable Effect Level.

c 35.

Soil samples were similarly highly contaminated at the mines and private properties (Fig. 4; Supplementary Table 1). The soil Pb concentrations exceeded the Welsh mineralization domain NBC (280 mg kg^−1^) at all sites except for Control 2, indicating that these sites contained soil Pb concentrations higher than those expected in mining areas in Wales (Supplementary Table 1) [36]. The Cd and Cu soil concentrations found at Mine 2 also exceeded the mineralization domain (Cd: 2.2 mg kg^−1^; Cu: 96 mg kg^−1^), as did the Cd concentrations at Mine Complex 1. While there are currently no NBCs for Zn, the Zn soil concentrations were above the ‘Sludge (Use in Agriculture) Regulations 1989’ concentration (200 mg kg^−1^) at all sites, with the exception of Control 2 (Supplementary Table 1) [37]. While the trace metal concentrations found at the control sites were lower than those found in the mine or private properties (Fig. 4), the soil samples from Control 1 had consistently higher trace metal concentrations than the soil samples from Control 2, particularly when sampling in locations around a particular dirt path.

### 3.2 Soil bioaccessibility

Following the BARGE extraction protocol to assess bioaccessible concentrations of the trace metals, the soil trace metal concentrations decreased by between 68.2 % and 96.1 % (Supplementary Table 1) [25,26]. However, the intestinal bioaccessible soil metal concentrations remained strongly correlated with the corresponding total soil metal concentrations for all four metals examined (Supplementary Fig. 1). Bioaccessibility percentages were generally low, especially for Zn and Pb, and particularly at the mine sites (where the mean percentages were 4.31 % and 7.41 %, respectively). Despite this, the mean Zn and Pb bioaccessibility concentrations at the mines (549 mg kg^−1^ and 451 mg kg^−1^, respectively) were still elevated above those found at the private properties and control sites, due to the high total metal concentrations found at the mine sites (Supplementary Table 1).

### 3.3 Invertebrates

**Fig. 5:**
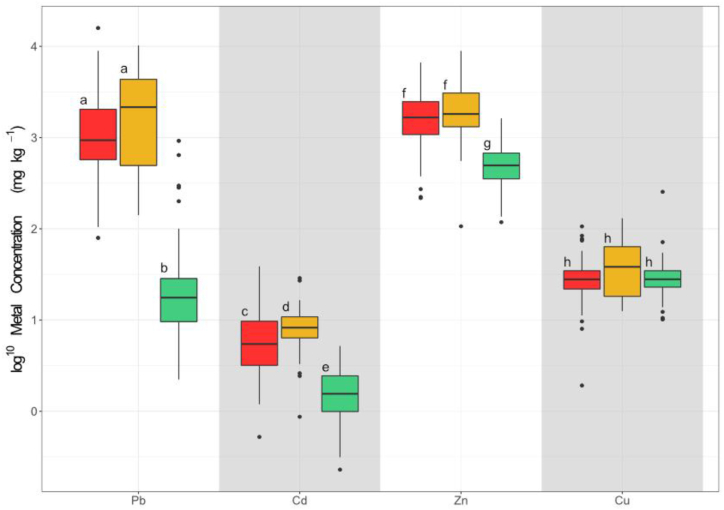
**Invertebrate log**^**10**^**transformed trace metal body burdens across mine, private property, and control sites.** Red represents the mine sites, yellow represents the private properties, and green represents the control sites. For each metal, site types with no letter in common are significantly different (pairwise Wilcox test; α = 5 % with Benjamini - Hochberg corrections).

Invertebrate full-body trace metal concentrations were highly variable across the sites (Supplementary Table 2). This was likely due to variations in inter-species trace metal accumulation, which significantly differed across families (Pb: df = 11, χ^2^ = 30.125, p = 0.00202; Cd: df = 11, χ^2^ = 36.9, p = 0.000478; Zn: df = 11, χ^2^ = 34.8, p = 0.000544; Cu: df = 11, χ^2^ = 20.4, p = 0.0404; Supplementary Fig. 2). When aggregated across families, invertebrate body burdens significantly varied across the mine, private property, and control sites for Pb (df = 2, χ^2^ = 114, p < 0.0001), Cd (df = 2, χ^2^ = 85.1, p < 0.0001), and Zn (df = 2, χ^2^ = 79.6, p < 0.0001) (Fig. 5). There were clear patterns of higher Pb, Cd, and Zn concentrations at the mine and private properties than at the control sites (Fig. 5; Supplementary Table 4). Lead, Cd, and Zn invertebrate body burdens were also strongly positively correlated with water concentrations (Pb: S [Spearman's Correlation Coefficient] = 163000, p < 0.0001, r_s_ = 0.770; Cd: S = 225000, p < 0.0001, r_s_ = 0.682; Zn: S = 209000, p < 0.0001, r_s_ = 0.705) and sediment (Pb: S = 175000, p < 0.0001, r_s_ = 0.743, Cd: S = 279000, p < 0.0001, r_s_ = 0.591; Zn: S = 258000, p < 0.0001, r_s_ = 0.622).

While Cu concentrations did not vary significantly across the three sites (df = 2, χ^2^ = 4.20, p = 0.122), the pattern of Cu invertebrate body burdens resembled that of Cu sediment concentrations, with the highest concentrations in the private properties, followed by the mine sites, and then the control sites (Fig. 4, Fig. 5). Invertebrate Cu body burdens were significantly correlated with sediment Cu concentrations (S = 493000, p = 0.000364, r_s_ = 0.278), though not with water concentrations (S = 612000, p = 0.0832).

### 3.4 Rodents

Four rodent species were collected during this study. Most of the rodents collected were wood mice (*Apodemus sylvaticus*, 111, 67.3 % of total), but yellow-necked mice (*Apodemus flavicollis*, 22, 13.3 % of total), field voles (*Microtus agrestis*, 18, 10.9 % of total), and bank voles (*Myodes glareolus*, 14, 8.48 %) were also found at the sites. Metal concentrations were found to be broadly statistically similar between species; however, to avoid species-specific variations in metal concentrations, which have been well documented in small mammals [[38], [39], [40], [41], [42]], only data on wood mice, the most frequently collected species, are presented below.

**Fig. 6:**
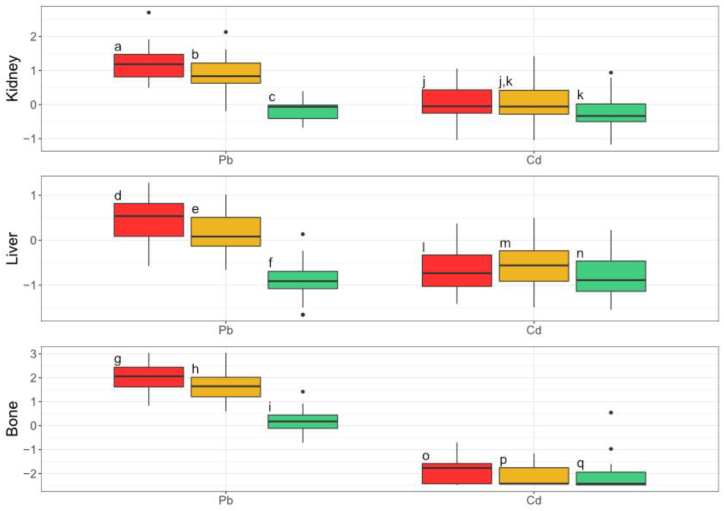
**Wood mouse log**^**10**^**transformed kidney, liver, and bone non-essential trace metal concentrations.** Red represents the mine sites, yellow represents the private properties, and green represents the control sites. For each metal and tissue, groups with no letter in common are significantly different (Tukey posthoc test; α = 5 % with Benjamini - Hochberg corrections).

The concentrations of the non-essential metals examined during this study (Pb and Cd) varied significantly between the mine, private property, and control sites in wood mouse kidney (Pb: df = 2, F = 103, p < 0.0001; Cd: df = 2, F = 6.25, p = 0.0329), liver (Pb: df = 2, F = 71.1, p < 0.0001; Cd: df = 2, F = 5.13, p = 0.0181), and, for Pb, in bone (Pb: df = 2, F = 112, p < 0.0001) (Supplementary Table 3). Across all three tissues, Pb concentrations tended to be greatest at the mine sites, followed by the private property, and finally by the control sites (Fig. 6; Supplementary Table 3). Cadmium in bone followed the same pattern, but mean Cd concentrations in kidney and liver were highest at the private properties, followed by the mine sites and then the control sites (Fig. 6; Supplementary Table 3). Wood mouse tissue Pb concentrations were strongly and significantly correlated with soil concentrations at the collection transects (kidney: df = 94, S = 45600, p > 0.0001, *ρ* = 0.690; liver: df = 107, S = 71700, p > 0.0001, *ρ* = 0.668, bone: df = 103, S = 67700, p > 0.0001, *ρ* = 0.649). Despite the significant differences in Cd across the site types, Cd concentrations did not significantly correlate with transect soil concentrations (kidney: df = 94, S = 133000, p = 0.449; liver: df = 107, S = 182000, p = 0.151; bone: df = 103, S = 152000, p = 0.0712).

**Fig. 7:**
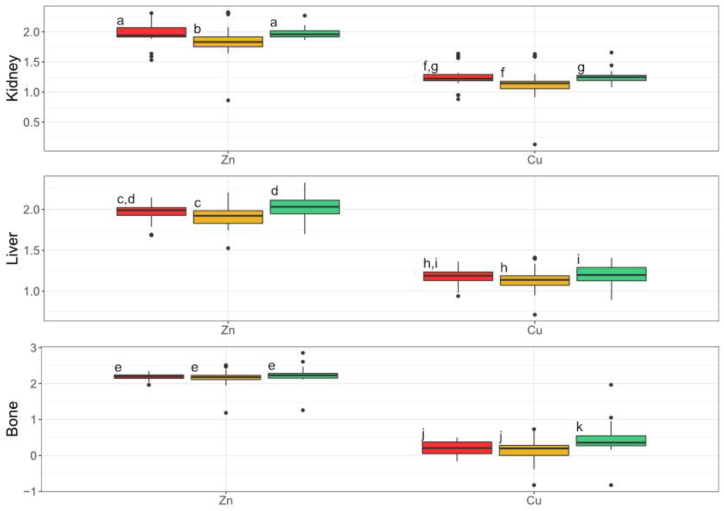
**Wood mouse log**^**10**^**transformed kidney, liver, and bone essential trace metal concentrations.** Red represents the mine sites, yellow represents the private properties, and green represents the control sites. For each metal and tissue, groups with no letter in common are significantly different (Tukey posthoc test; α = 5 % with Benjamini - Hochberg corrections).

The concentrations of the essential metals examined during this study, Zn and Cu, also varied significantly across the mine, private property, and control sites in wood mice kidneys (Zn: df = 2, F = 7.60, p = 0.00324; Cu: df = 2, F = 6.28, p = 0.00796) and livers (Zn: df = 2, F = 11.0, p = 0.000294; Cu: df = 2, F = 6.05, p = 0.00846), as well as in bone for Cu df = 2, F = 8.86, p = 0.00137) (Supplementary Table 4). However, the trends across the site types completely differed from those observed for Pb and Cd. The Zn and Cu kidney concentrations were highest in the mine sites, followed by the control sites, and then the private properties, while the liver Zn and Cu concentrations were highest in the control sites, then private properties, and then mine sites, and the bone Zn and Cu concentrations were highest in the control sites, followed by the mine sites, and then the private properties (Fig. 7). The only significant correlation detected between the essential elements (Zn and Cu) across soil transects and wood mouse tissues was an inverse relationship between Cu concentrations in soil and wood mouse bones (df = 103, S = 251000, p = 0.00503, *ρ* = −0.303).

Beyond site type, trace metal concentrations varied depending on a range of factors (Supplementary Table 4). In particular, the collection time (month and year) significantly affected concentrations of every trace metal and tissue, apart from Cu in bones and Cd in kidneys (Supplementary Tables 4–5). The mean concentrations of Pb and Cd in kidney and liver tissues were similar across the collection months, though with greater variation in May 2019 for Cd and October 2019 for Pb, while mean Pb and Cd bone concentrations were highest in September 2021 (Supplementary Fig. 3; Supplementary Table 5). For the essential metals, concentrations were consistently lower in May 2019 than in October 2019 across all three tissues, though concentrations in September 2021 varied depending on the metal and tissue (Supplementary Fig. 3; Supplementary Table 5). Other factors that influenced trace metal concentrations were whether the wood mouse was collected in Area 1 or Area 2 (there were significant differences across all metals and tissues, except for Cd in bones and Zn in kidneys and bones) and whether the wood mouse was a juvenile or adult (there were significant differences across all metals in kidneys, Zn and Cu in livers, and Pb in bones) (Supplementary Tables 4–5). Factors that did not appear to greatly affect trace metal concentrations included the sex of the wood mouse (only significant differences were between Cd concentrations in kidneys and in livers) and the individual site in which the wood mouse was collected (only significant differences were between Cd concentrations in kidneys and in bones) (Supplementary Tables 4–5).

## 4 Discussion

### 4.1 Trace metal contamination distribution

Notably high and potentially toxic concentrations of Pb, Cd, Zn, and Cu were found in the water, sediment, and soils of the two derelict mine sites, as well as the two private properties located 1 km and 4 km, respectively, downstream of the mine sites. While abandoned spoil heaps provided a clear and ongoing source of trace metal pollution at the mine sites, the private properties appeared visually pristine, without any obvious signs of trace metal contamination. However, as the mine sites have likely been operational on and off since pre-Roman times, the areas downstream of the mines have been accumulating trace metals for thousands of years, likely leading to the high concentrations detected during this study. The high metal concentrations found at the private properties may be due to a combination of continuous spoil heap water erosion, aerial deposition of particulates from the spoil heaps [[43], [44], [45]], and past flooding events [[46], [47], [48]]. Additional anthropogenic activities can further distribute trace metal particulates; for example, both property owners reported the frequent use of recreational off-road vehicles at the mine sites, which can generate airborne dust and mobile tailing particulates [44].

Anthropogenic activities can also more directly cause the spread of trace metals beyond the mine sites. In the surveyed region, it is common to utilize mine waste as gravel for roads or tracks, due to its easy availability [49]. It appears that a path in Control 1 could have contained this material, as gravel excavated from this path had a Pb concentration (7750 ± 545 mg kg^−1^) that resembled the levels found in the mine spoil in Mine 1b. While this path has now mostly been covered by soil, samples collected from rodent transects near this path had notably elevated Pb soil concentrations (2410 ± 1790 mg kg^−1^). Despite this, the wood mouse Pb tissue concentrations sampled at this site did not significantly differ with those found at Control 2 (kidney: t = −1.80, p = 0.276; liver: t = 0.0704, p = 0.945; bone: t = −0.508, p = 0.935). In this instance, it appears that the high Pb soil concentrations were not transferring into local rodents, possibly due to the fact the contaminated gravel was covered with soil, along with the lack of vegetative cover and, therefore, poor foraging opportunities on the path. However, it is still important to note that the widespread use of mine spoil as gravel can spread mine waste beyond the original mine site and even to otherwise uncontaminated areas.

### 4.2 Trace metal transfer into wildlife

Aquatic invertebrates collected at the mine sites and downstream private properties had significantly higher Pb, Cd, and Zn body burdens than invertebrates from the control sites. Trace metal accumulation patterns varied across families, as expected, as metal tolerance in invertebrates is well known to vary greatly across taxonomic groups [4], but these differences did not mask the effects of exposure to high trace metal concentrations. Other studies have similarly observed elevated concentrations of these trace metals in aquatic invertebrates in metal-contaminated environments [4,[50], [51], [52], [53], [54], [55], [56]]. While the reported concentrations may be elevated above the true invertebrate tissue concentrations, as the gut contents of the invertebrates were not purged (to allow for a better estimate of predator trace metal exposure), many of the invertebrate concentrations were higher than their corresponding sediment concentrations, suggesting metal accumulation. The invertebrate Pb, Cd, and Zn body burdens were also strongly correlated with the water and sediment concentrations, as previously observed for freshwater macroinvertebrates [52]. Unlike the other metals, however, invertebrate Cu concentrations did not significantly differ across site types or correlate with water concentrations, though they did correlate with Cu sediment concentrations. This is consistent with the relatively low Cu contamination at all sampled sites.

Similar to the macroinvertebrates, wood mice collected from contaminated sites accumulated significantly more Pb and Cd than wood mice from the control sites, as has been observed in previous studies [[57], [58], [59], [60], [61]]. While other factors, such as location, season, and age, affected non-essential metal exposure, as expected [58,60,[62], [63], [64]], the concentrations of non-essential trace metals in the environment did have an effect on trace metal tissue concentrations in wood mice.

Pb concentrations in wood mouse tissues were strongly positively correlated with Pb soil concentrations. Lead bioaccessibility in the sampled soils, particularly at the mine sites, was generally low, which is consistent with prior studies on Pb-contaminated soils [59,65], especially at Pb mine sites [[66], [67], [68]]. Although bioaccessibility was assessed during this study using a simulated human gut system, the murine gastrointestinal tract is broadly similar to those of humans, with some exceptions [69], so this model should provide a reasonable approximation of bioaccessibility to rodents. Despite the low bioaccessibility of Pb in the soil, the high total concentrations of Pb in the mines and private properties meant that rodents were still exposed to high concentrations of bioaccessible Pb, resulting in Pb accumulation within their tissues [70].

While toxicity thresholds are difficult to generate for trace metals, there have been a few suggested toxicity thresholds for Pb in rodents, primarily linked to observations of potentially Pb associated health effects in the field. Based on Shore and Douben's [71] proposed Pb toxic threshold for small mammals (25 mg kg^−1^ d.w. in kidneys), ten individuals (nine wood mice and one bank vole) were at risk of Pb toxicity (seven from Mine 2 and three from Private Property 2, again all collected in Area 2) [72]. In a review of the existing literature, Ma [14] suggested that mammalian kidney Pb concentrations greater than 15 mg kg^−1^ d.w., and bone Pb concentrations greater than 25 mg kg^−1^ d.w., can be associated with kidney damage. Based on Ma's [14] kidney threshold, 28 individuals (26 wood mice, one yellownecked mouse, and one bank vole), 14 each at the mines and private properties, were at potential risk of kidney damage. Ma's [14] proposed bone Pb threshold was exceeded by 38 individuals (93 % of the collected rodents) at the mine sites, and 51 individuals (76 % of the collected rodents) at the private properties.

As was found for Pb, Cd kidney and liver concentrations were elevated in wood mice from the mine sites and private properties when compared to the control sites. This agrees with prior studies on wood mice in Cd-polluted sites, which have found elevated kidney and liver Cd concentrations, but little Cd accumulation in bones [57,59]. While most studies in contaminated sites have reported rodent Cd tissue concentrations higher than those observed in the current study [38,59,61,64,73], this finding was expected, as mine sites in western Wales are known to have relatively low soil Cd concentrations [63]. As such, the wood mouse Cd concentrations observed during this study were uniformly below Shore & Douben's [39] proposed threshold for kidney damage in small mammals of 105 mg kg^−1^ d.w.

In contrast to the accumulation patterns observed with the non-essential elements, the concentrations of essential elements (Cu and Zn) in wood mice examined during the current study did not appear to be affected by local contamination. This has been reported in previous studies on rodents, and researchers have suggested that the observed lack of Cu and Zn accumulation is due to the tight biological regulation of essential elements [38,59,72,74,[75], [76]]. While some studies have found elevated Cu or Zn rodent tissue concentrations in highly contaminated sites [38,59], wood mice generally do not bioconcentrate Cu or Zn in kidneys or livers, even when fed high-Zn diets [38,62]. In fact, inverse relationships between environmental contamination and Cu or Zn concentrations, as observed during the current study between Cu in soil and in wood mouse bones, have previously been found for Zn in wood mice [74] and bank voles [77] collected near mines in west Wales, and for Cu in shrews in Poland [75]. One possible explanation could be that rodents in contaminated areas modify their diets to avoid high concentrations of Cu and Zn, which has been observed in both laboratory experiments [78] and field studies [79]. Another potential explanation is that rodents exposed to high concentrations of dietary Zn may reduce Zn absorption (through synthesis of metallothionein proteins, triggered by the high Zn concentrations) and increase their Zn excretion rate, therefore decreasing overall Zn accumulation [74].

The concentrations of Cu and Zn in wood mouse tissues appeared to be more influenced by season than by the contamination gradient, based on the consistent increases in Cu and Zn concentrations from May to October 2019 (Supplementary Fig. 3). Prior studies have found indications that rodent Cu and Zn tissue concentrations vary at least partially based on season [40,80,81]. These differences are most likely attributable to seasonal changes in diet [82], which are well-recorded in wood mice [83] and have been known to affect seasonal Cu and Zn exposure in rodents [80].

### 4.3 Individual and ecosystem health implications

Chronic exposure to high trace metal concentrations can have a variety of adverse health effects. In aquatic invertebrates, trace metal exposure is well known to result in increased mortality, affecting community structures and often causing full extirpation of species from metal-contaminated environments [4,[84], [85], [86], [87]]. Trace metals can also cause a variety of sublethal health effects, impacting invertebrate oviposition, hatching, development, emergence, growth, locomotion, and feeding [4,85,88].

Rodents can be similarly impacted by living in environments contaminated by trace metals. In mammals, chronic exposure to high Pb concentrations can have negative health effects across multiple physiological systems, including the central nervous system, renal system, hematopoietic system, the cardiovascular system, and the gastrointestinal system [14]. Measuring chronic negative health effects in short-lived species, such as rodents, is difficult, but a few studies have successfully linked environmental trace metal exposure with histological abnormalities [14,61,89]. Based on the concentrations found in those studies, it is possible that the rodents collected during the current study had similar histological abnormalities.

Determining the exposure to, and accumulation of, trace metals in common prey species, such as aquatic invertebrates and rodents, is the first step in understanding trace metal accumulation and impacts across all resident fauna. While trace metals do not traditionally biomagnify, they can transfer directly from prey to predator. For example, earthworms can accumulate high concentrations of Cd, which can then transfer to small mammals, who consequently develop higher Cd tissue concentrations than is found in their surrounding environment [41]. Furthermore, even if trace metal biomagnification is limited, invertebrates and rodents can serve as trace metal biomonitors to assess relative environmental contamination and to determine the potential exposure risks for other resident animals [4,85,90]. If aquatic invertebrates and rodents are accumulating high, potentially toxic concentrations of trace metals in their tissues, it is reasonable to suspect that other local animals are doing so as well. In this way, these prey species can act as ‘canaries in the coal mine’, signalling the ecosystem-wide effects of trace metal pollution [4,41,60,90].

### 4.4 Conclusions

Trace metal pollution can pose a serious threat to ecosystem health. While the former metalliferous mines examined during the current study have been closed for over a hundred years, the legacy of trace metal pollution can still be found throughout the local ecosystem. High concentrations of Pb, Zn, and, to a lesser extent, Cd and Cu, were found at both the mine sites and at properties several km downstream of the mines, in areas that appeared visually pristine. The trace metal concentrations found in the water and sediments of the streams flowing through the mine sites were sufficiently elevated that they could pose a health risk to local aquatic animals. High concentrations of Pb, Cd, Zn, and Cu were also found in aquatic invertebrates living in these streams. These elevated invertebrate trace metal body burdens were directly correlated with the concentrations of these metals in the water (for all but Cu) and in the sediment at their collection sites. On land, although wood mice appeared able to regulate the concentrations of the essential elements (Zn and Cu) that they were exposed to, they accumulated high concentrations of Pb, positively related to local Pb soil concentrations. While there are currently no clear Pb health thresholds for rodents, the Pb concentrations may be indicative of kidney damage in between 10 % and 82 % of the rodents collected at both the mine sites and areas downstream. The wood mice at the mine sites and downstream private properties also had elevated Cd tissue concentrations when compared to wood mice from control sites, though the Cd concentrations were not elevated above current suggested health thresholds. Overall, when assessing metal pollution, full ecosystem surveys encompassing areas beyond the pollution's origin point are necessary to assess potential adverse impacts across multiple trophic levels. Globally, there are hundreds of thousands, if not millions, of abandoned or currently active metal mines, and the continued high demand for metals will encourage further metal mining in the future. Furthermore, more frequent flooding and storms as a result of climate change will mobilise and re-distribute trace metal pollutants already present in the environment, exacerbating their reach and adverse impact. A better understanding of the environmental distribution of trace metal pollution, its transfer to local fauna, and the resultant health impact is crucially required to more effectively detect, manage, and mitigate the impacts of these global pollutants.

## Funding details

This work was supported by the 10.13039/501100000270Natural Environment Research Council [NERC grant reference number 10.13039/100006147NE/L002604/1], with Andrea Sartorius's studentship through the Envision Doctoral Training Partnership. Further funding for this project was provided by 10.13039/100009261Natural Resources Wales and the 10.13039/501100000837University of Nottingham.

## Data availability

The data that support the findings of this study are available upon request.

The Natural Resources Wales data used for dissolved organic carbon concentrations is publicly available and contains public sector information licensed under the Open Government Licence v3.0.

## CRediT authorship contribution statement

**Andrea Sartorius:** Writing – review & editing, Writing – original draft, Visualization, Investigation, Funding acquisition, Formal analysis, Conceptualization. **Matthew Johnson:** Writing – review & editing, Supervision, Resources, Methodology, Investigation, Conceptualization. **Scott Young:** Writing – review & editing, Supervision, Resources. **Malcolm Bennett:** Writing – review & editing, Supervision, Resources, Methodology, Investigation. **Kerstin Baiker:** Supervision, Methodology, Investigation. **Paul Edwards:** Writing – review & editing, Resources. **Lisa Yon:** Writing – review & editing, Supervision, Investigation, Funding acquisition, Conceptualization.

## Declaration of competing interest

The authors declare that they have no known competing financial interests or personal relationships that could have appeared to influence the work reported in this paper.

## References

[1] Hong S., Candelone J.P., Patterson C.C., Boutron C.F. (1996). History of ancient copper smelting pollution during Roman and medieval times recorded in Greenland ice. Science.

[2] Hernberg S. (2000). Lead poisoning in a historical perspective. Am. J. Ind. Med..

[3] Johnson D.B. (2003). Chemical and microbiological characteristics of mineral spoils and drainage waters at abandoned coal and metal mines. Water Air Soil Pollut. Focus.

[4] Rainbow P.S. (2018).

[5] Davies B.E. (1987). Ecological Effects of in Situ Sediment Contaminants.

[6] Nicholson F.A., Smith S.R., Alloway B.J., Carlton-Smith C., Chambers B.J. (2003). An inventory of heavy metals inputs to agricultural soils in England and Wales. Sci. Total Environ..

[7] Alhar M.A., Thompson D.F., Oliver I.W. (2021). Mine spoil remediation via biochar addition to immobilise potentially toxic elements and promote plant growth for phytostabilisation. J. Environ. Manag..

[8] Jentsch A., Beierkuhnlein C. (2008). Research frontiers in climate change: effects of extreme meteorological events on ecosystems. Compt. Rendus Geosci..

[9] Hirabayashi Y., Mahendran R., Koirala S., Konoshima L., Yamazaki D., Watanabe S., Kim H., Kanae S. (2013). Global flood risk under climate change. Nat. Clim. Change.

[10] Pyatt F.B., Gilmore G., Grattan J.P., Hunt C.O., Mclaren S. (2000). An imperial legacy? An exploration of the environmental impact of ancient metal mining and smelting in southern Jordan. J. Archaeol. Sci..

[11] Wilson B., Pyatt F.B. (2007). Heavy metal dispersion, persistance, and bioccumulation around an ancient copper mine situated in Anglesey, UK. Ecotoxicol. Environ. Saf..

[12] Hunter B.A., Johnson M.S. (1982). Food chain relationships of copper and cadmium in contaminated grassland ecosystems. Oikos.

[13] Heikens A., Peijnenburg W.J.G.M., Hendriks A.J. (2001). Bioaccumulation of heavy metals in terrestrial invertebrates. Environ. Pollut..

[14] Ma W.C. (2011). Environmental Contaminants in Biota.

[15] Gall J.E., Boyd R.S., Rajakaruna N. (2015). Transfer of heavy metals through terrestrial food webs: a review. Environ. Monit. Assess..

[16] Kalisińska E. (2019). Mammals and Birds as Bioindicators of Trace Element Contaminations in Terrestrial Environments: an Ecotoxicological Assessment of the Northern Hemisphere.

[17] Tchounwou P.B., Yedjou C.G., Patlolla A.K., Sutton D.J. (2012). Molecular, Clinical, and Environmental Toxicology.

[18] Her Majesty’s Stationary Office (1985). Methods of biological handnet sampling of aquatic benthic macroinvertebrates. Methods for the Examination of Waters and Associated Materials.

[19] Environment Agency (2009).

[20] Montgomery W.I., Dowie M. (1993). The distribution and population regulation of the wood mouse *Apodemus sylvaticus* on field boundaries of pastoral farmland. J. Appl. Ecol..

[21] Gurnell J. (1978). Seasonal changes in numbers and male behavioural interaction in a population of wood mice, *Apodemus sylvaticus*. J. Anim. Ecol..

[22] Green R. (1979). The ecology of wood mice (*Apodemus sylvaticus*) on arable farmland. J. Zool..

[23] Marin Ş., Lăcrimioara Ş., Cecilia R. (2011). Evaluation of performance parameters for trace elements analysis in perennial plants using ICP-OES technique. J. Plant Dev..

[24] Kushner E.J. (1976). On determining the statistical parameters for pollution concentration from a truncated data set. Atmos. Environ..

[25] Wragg J., Cave M., Taylor H., Basta N., Brandon E., Casteel S., Gron C., Oomen A., Van de Wiele T. (2009). Inter-laboratory trial of a unified bioaccessibility testing procedure. Nottingham, UK. British Geological Survey.

[26] BARGE, & INERIS (2010). UBM Procedure for the Measurement of Inorganic Contaminant Bioaccessibility from Solid Matrices.

[27] Ma W.C. (1989). Effect of soil pollution with metallic lead pellets on lead bioaccumulation and organ/body weight alterations in small mammals. Arch. Environ. Contam. Toxicol..

[28] R Core Team (2018). https://www.R-project.org/.

[29] Sparks T. (2000).

[30] Wickham H. (2016).

[31] Benjamini Y., Hochberg Y. (1995). Controlling the false discovery rate: a practical and powerful approach to multiple testing. J. Roy. Stat. Soc. B.

[32] Yekutieli D., Benjamini Y. (1999). Resampling-based false discovery rate controlling multiple test procedures for correlated test statistics. J. Stat. Plann. Inference.

[33] Defra (2014).

[34] Water Framework Directive United Kingdom Technical Advisory Group (2014). https://www.wfduk.org/sites/default/files/Media/Environmentalstandards/MBATUKTAGMethodStatement.pdf.

[35] Canadian Council of Ministers of the Environment (2001). *Canadian Environmental Quality Guidelines*, 1999.

[36] Ander E.L., Cave M.R., Johnson C.C. (2013). http://nora.nerc.ac.uk/id/eprint/501566.

[37] Public Health, England and Wales, & Public Health, Scotland (1989).

[38] Talmage S.S., Walton B.T. (1991). Small mammals as monitors of environmental contaminants. Rev. Environ. Contam. Toxicol..

[39] Shore R.F., Douben P.E. (1994). The ecotoxicological significance of cadmium intake and residues in terrestrial small mammals. Ecotoxicol. Environ. Saf..

[40] Wijnhoven S., Leuven R.S.E.W., van der Velde G., Jungheim G., Koelemij E.I., De Vries F.T., Eijsackers H.J.P., Smits A.J.M. (2007). Heavy-metal concentrations in small mammals from a diffusely polluted floodplain: importance of species-and location-specific characteristics. Arch. Environ. Contam. Toxicol..

[41] Cooke J.A. (2011). Environmental Contaminants in Biota: Interpreting Tissue Concentrations.

[42] Van den Brink N.W., Lammertsma D.R., Dimmers W.J., Boerwinkel M.C. (2011). Cadmium accumulation in small mammals: species traits, soil properties, and spatial habitat use. Environ. Sci. Technol..

[43] Moreno T., Oldroyd A., McDonald I., Gibbons W. (2007). Preferential fractionation of trace metals–metalloids into PM10 resuspended from contaminated gold mine tailings at Rodalquilar, Spain. Water Air Soil Pollut..

[44] Corriveau M.C., Jamieson H.E., Parsons M.B., Campbell J.L., Lanzirotti A. (2011). Direct characterization of airborne particles associated with arsenic-rich mine tailings: particle size, mineralogy and texture. Appl. Geochem..

[45] Martín-Crespo T., Gómez-Ortiz D., Martín-Velázquez S., Martínez-Pagán P., de Ignacio-San José C., Lillo J., Faz Á. (2020). Abandoned mine tailings affecting riverbed sediments in the cartagena–La union district, mediterranean coastal area (Spain). Rem. Sens..

[46] Middelkoop H. (2000). Heavy-metal pollution of the river Rhine and Meuse floodplains in The Netherlands. Neth. J. Geosci..

[47] Foulds S.A., Brewer P.A., Macklin M.G., Haresign W., Betson R.E., Rassner S.M.E. (2014). Flood-related contamination in catchments affected by historical metal mining: an unexpected and emerging hazard of climate change. Sci. Total Environ..

[48] Marrugo-Negrete J., Pinedo-Hernández J., Díez S. (2017). Assessment of heavy metal pollution, spatial distribution and origin in agricultural soils along the Sinú River Basin, Colombia. Environ. Res..

[49] Sartorius A., Johnson M., Young S., Bennett M., Baiker K., Edwards P., Yon L. (2022). Human health implications from consuming eggs produced near a derelict metalliferous mine: a case study. Food Addit. Contam..

[50] Spehar R.L., Anderson R.L., Fiandt J.T. (1978). Toxicity and bioaccumulation of cadmium and lead in aquatic invertebrates. Environ. Pollut..

[51] Eisler R. (1988). https://pubs.er.usgs.gov/publication/5200021.

[52] Goodyear K.L., McNeill S. (1999). Bioaccumulation of heavy metals by aquatic macro-invertebrates of different feeding guilds: a review. Sci. Total Environ..

[53] Solà C., Prat N. (2006). Monitoring metal and metalloid bioaccumulation in Hydropsyche (Trichoptera, Hydropsychidae) to evaluate metal pollution in a mining river. Whole body versus tissue content. Sci. Total Environ..

[54] Santoro A., Blo G., Mastrolitti S., Fagioli F. (2009). Bioaccumulation of heavy metals by aquatic macroinvertebrates along the Basento River in the south of Italy. Water Air Soil Pollut..

[55] De Jonge M., Tipping E., Lofts S., Bervoets L., Blust R. (2013). The use of invertebrate body burdens to predict ecological effects of metal mixtures in mining-impacted waters. Aquat. Toxicol..

[56] Arnold A., Murphy J.F., Pretty J.L., Duerdoth C.P., Smith B.D., Rainbow P.S., Spencer K.L., Collins A.L., Jones J.I. (2021). Accumulation of trace metals in freshwater macroinvertebrates across metal contamination gradients. Environ. Pollut..

[57] Johnson M.S., Roberts R.D., Hutton M., Inskip M.J. (1978). Distribution of lead, zinc and cadmium in small mammals from polluted environments. Oikos.

[58] Milton A., Johnson M.S., Cooke J.A. (2002). Lead within ecosystems on metalliferous mine tailings in Wales and Ireland. Sci. Total Environ..

[59] Rogival D., Scheirs J., Blust R. (2007). Transfer and accumulation of metals in a soil–diet–wood mouse food chain along a metal pollution gradient. Environ. Pollut..

[60] Sánchez-Chardi A., Peñarroja-Matutano C., Ribeiro C.A.O., Nadal J. (2007). Bioaccumulation of metals and effects of a landfill in small mammals. Part II. The wood mouse. Apodemus sylvaticus. Chemosphere.

[61] Tête N., Durfort M., Rieffel D., Scheifler R., Sánchez-Chardi A. (2014). Histopathology related to cadmium and lead bioaccumulation in chronically exposed wood mice, Apodemus sylvaticus, around a former smelter. Sci. Total Environ..

[62] Hunter B.A., Johnson M.S., Thompson D.J. (1987). Ecotoxicology of copper and cadmium in a contaminated grassland ecosystem. III. Small mammals. J. Appl. Ecol..

[63] Milton A., Cooke J.A., Johnson M.S. (2004). A comparison of cadmium in ecosystems on metalliferous mine tailings in Wales and Ireland. Water Air Soil Pollut..

[64] Tomza-Marciniak A., Pilarczyk B., Marciniak A., Udała J., Bąkowska M., Pilarczyk R. (2019). Mammals and Birds as Bioindicators of Trace Element Contaminations in Terrestrial Environments.

[65] Bosso S.T., Enzweiler J. (2008). Bioaccessible lead in soils, slag, and mine wastes from an abandoned mining district in Brazil. Environ. Geochem. Health.

[66] Rieuwerts J.S., Farago M.E., Cikrt M., Bencko V. (2000). Differences in lead bioavailability between a smelting and a mining area. Water Air Soil Pollut..

[67] Hettiarachchi G.M., Pierzynski G.M. (2004). Soil lead bioavailability and in situ remediation of lead‐contaminated soils: a review. Environ. Prog..

[68] Intawongse M., Dean J.R. (2006). In-vitro testing for assessing oral bioaccessibility of trace metals in soil and food samples. TrAC, Trends Anal. Chem..

[69] Nguyen T.L.A., Vieira-Silva S., Liston A., Raes J. (2015). How informative is the mouse for human gut microbiota research?. Disease models & mechanisms.

[70] Baranowska-Bosiacka I., Korbecki J., Marchlewicz M. (2019). Mammals and Birds as Bioindicators of Trace Element Contaminations in Terrestrial Environments.

[71] Shore R.F., Douben P.E. (1994). Predicting ecotoxicological impacts of environmental contaminants on terrestrial small mammals. Rev. Environ. Contam. Toxicol..

[72] Fritsch C., Cosson R.P., Cœurdassier M., Raoul F., Giraudoux P., Crini N., De Vaufleury A., Scheifler R. (2010). Responses of wild small mammals to a pollution gradient: host factors influence metal and metallothionein levels. Environ. Pollut..

[73] Shore R.F. (1995). Predicting cadmium, lead and fluoride levels in small mammals from soil residues and by species-species extrapolation. Environ. Pollut..

[74] Milton A., Johnson M.S. (2002). Food chain transfer of zinc within the ecosystems of old and modern metalliferous mine wastes. Environ. Technol..

[75] Świergosz‐Kowalewska R., Gramatyka M., Reczyński W. (2005). Metals distribution and interactions in tissues of shrews (*Sorex spp.*) from copper‐and zinc‐contaminated areas in Poland. J. Environ. Qual..

[76] Martiniaková M., Omelka R., Stawarz R., Formicki G. (2012). Accumulation of lead, cadmium, nickel, iron, copper, and zinc in bones of small mammals from polluted areas in Slovakia. Pol. J. Environ. Stud..

[77] Milton A., Cooke J.A., Johnson M.S. (2003). Accumulation of lead, zinc, and cadmium in a wild population of *Clethrionomys glareolus* from an abandoned lead mine. Arch. Environ. Contam. Toxicol..

[78] Beernaert J., Scheirs J., Van Den Brande G., Leirs H., Blust R., De Meulenaer B., Van Camp J., Verhagen R. (2008). Do wood mice (*Apodemus sylvaticus* L.) use food selection as a means to reduce heavy metal intake?. Environ. Pollut..

[79] Ozaki S., Fritsch C., Valot B., Mora F., Cornier T., Scheifler R., Raoul F. (2018). Does pollution influence small mammal diet in the field? A metabarcoding approach in a generalist consumer. Mol. Ecol..

[80] Włostowski T., Chętnicki W., Gierłachowska-Bałdyga W., Chycak B. (1988). Zinc, iron, copper, manganese, calcium and magnesium supply status of free-living bank voles. Acta Theriol..

[81] Demir F.T., Yavuz M. (2020). Heavy metal accumulation and genotoxic effects in levant vole (*Microtus guentheri*) collected from contaminated areas due to mining activities. Environ. Pollut..

[82] Eisler R. (1993). https://pubs.er.usgs.gov/publication/5200116.

[83] Montgomery S.S.J., Montgomery W.I. (1990). Intrapopulation variation in the diet of the wood mouse. Apodemus sylvaticus. Journal of Zoology.

[84] Dickman M.D., Yang J.R., Brindle I.D. (1990). Impacts of heavy metals on higher aquatic plant, diatom and benthic invertebrate communities in the Niagara River watershed near Welland, Ontario. Water Quality Research Journal.

[85] Hare L. (1992). Aquatic insects and trace metals: bioavailability, bioaccumulation, and toxicity. Crit. Rev. Toxicol..

[86] Clements W.H. (1994). Benthic invertebrate community responses to heavy metals in the Upper Arkansas River Basin, Colorado. J. North Am. Benthol. Soc..

[87] Maret T.R., Cain D.J., MacCoy D.E., Short T.M. (2003). Response of benthic invertebrate assemblages to metal exposure and bioaccumulation associated with hard-rock mining in northwestern streams, USA. J. North Am. Benthol. Soc..

[88] Di Veroli A., Santoro F., Pallottini M., Selvaggi R., Scardazza F., Cappelletti D., Goretti E. (2014). Deformities of chironomid larvae and heavy metal pollution: from laboratory to field studies. Chemosphere.

[89] Roberts R.D., Johnson M.S., Hutton M. (1978). Lead contamination of small mammals from abandoned metalliferous mines. Environ. Pollut..

[90] Al Sayegh Petkovšek S., Kopušar N., Kryštufek B. (2014). Small mammals as biomonitors of metal pollution: a case study in Slovenia. Environ. Monit. Assess..

