# Supplementary material for: Trace metal accumulation through the environment and wildlife at two derelict lead mines in Wales
Source: Heliyon. 2024 Jul 9;10(14):e34265. doi: 10.1016/j.heliyon.2024.e34265 (PMC11284397; doi:10.1016/j.heliyon.2024.e34265)
Supplement: Multimedia component 1 [file mmc1.docx]

## Appendices

### Appendix A: Supplementary Figures


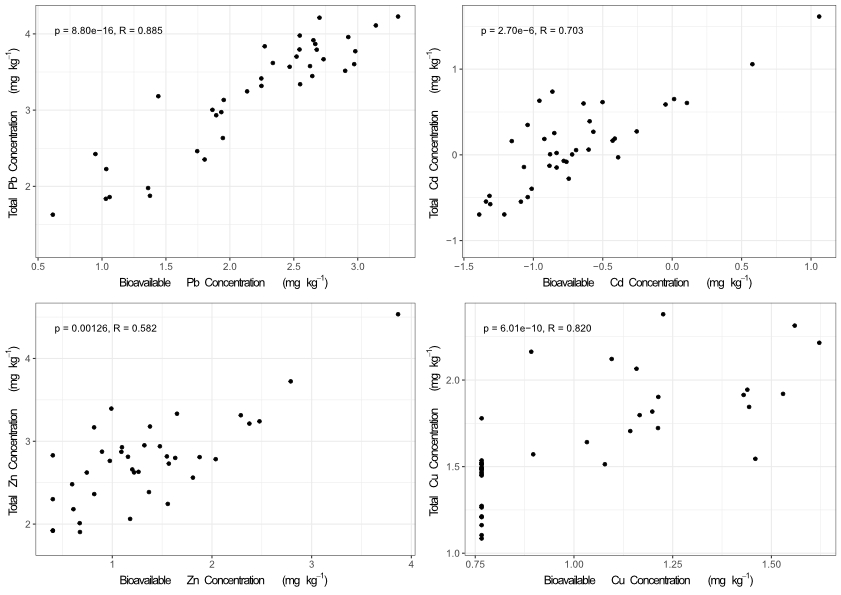


**Supplementary Figure 1: Total versus bioaccessible soil trace metal concentrations.**

Spearman’s correlation coefficients are shown in the top right; the p-values are adjusted

following Yekutieli & Benjamini (1999).


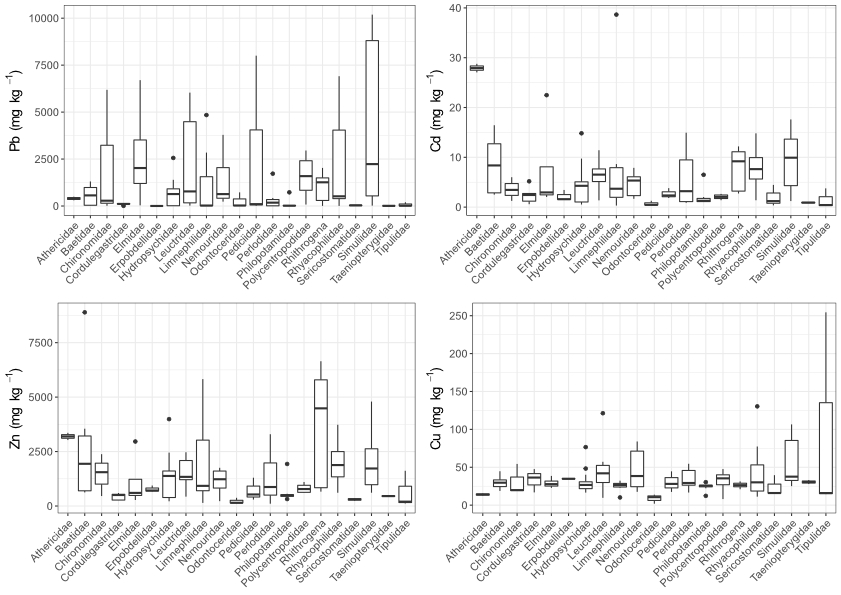


**Supplementary Figure 2: Pb, Cd, Zn, and Cu concentrations across collected invertebrate families at all sampled sites.**


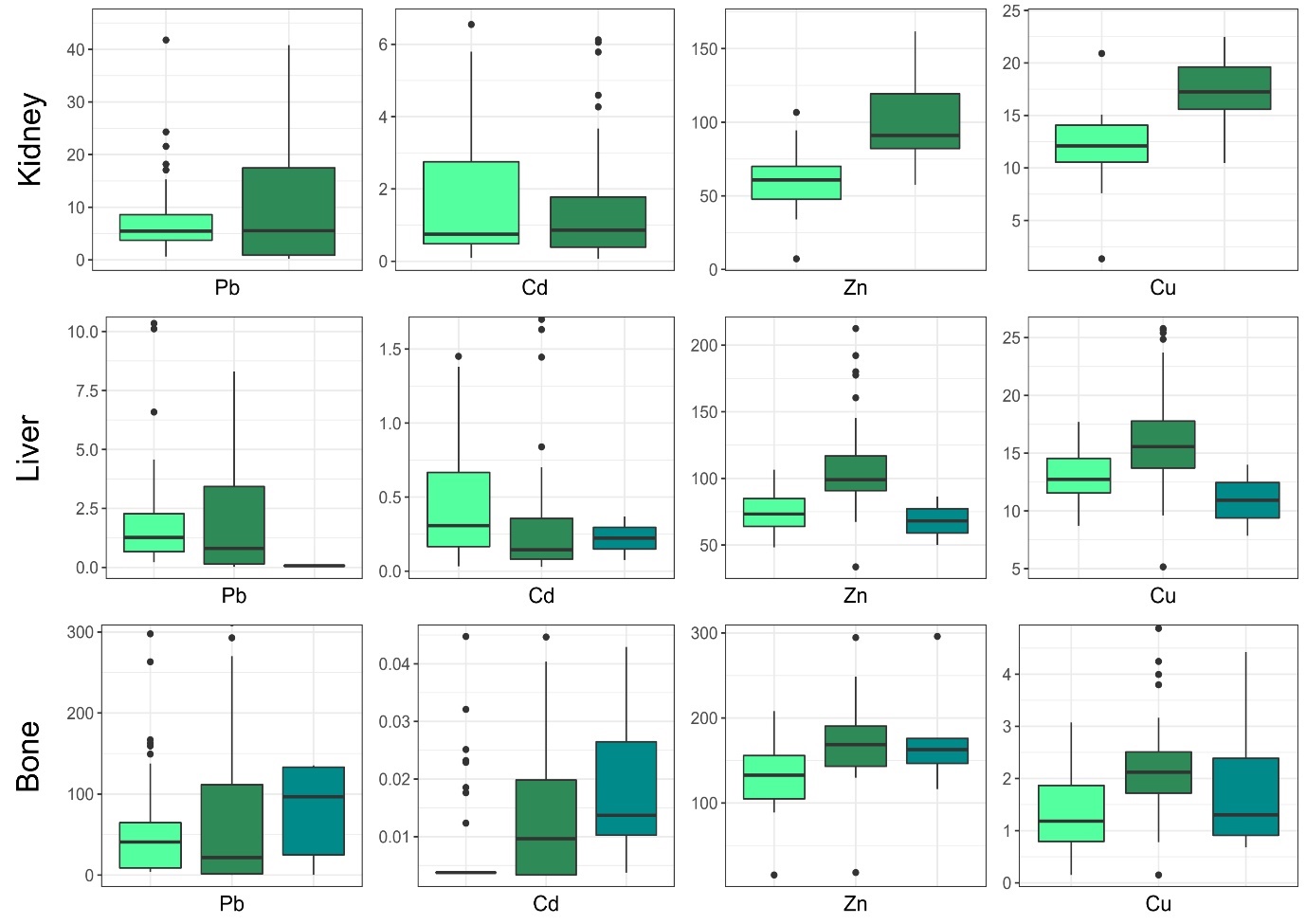


**Supplementary Figure 3: Wood mouse kidney, liver, and bone trace metal concentrations across collection month.** Rodents collected in May 2019 are on the left, in light green, rodents collected in October 2019 are on the middle, in dark green, and rodents collected in September 2021 are on the right in blue.

### Appendix B: Supplementary Tables

**Supplementary Table 1: Mean total and bioaccessible soil trace metal concentrations across the mine, private property, and control sites.** Means are reported as ± standard deviation. Concentrations are in mg kg^-1^.

| **Site** |  | **Pb** | **Cd** | **Zn** | **Cu** |
| --- | --- | --- | --- | --- | --- |
| Mine | Total | 6100 ± 4950 | 5.58 ± 10.2 | 3550 ± 8570 | 80.5 ± 59.8 |
|  | Bioaccessible | 451 ± 506 | 1.17 ± 2.99 | 549 ± 1890 | 14.1 ± 9.76 |
|  | Bioaccessible % | 7.41 ± 4.04 | 13.2 ± 9.81 | 4.31 ± 5.62 | 19.5 ± 7.73 |
| Private Property | Total | 4115 ± 3880 | 1.34 ± 1.06 | 596 ± 486 | 67.6 ± 54.5 |
|  | Bioaccessible | 400 ± 396 | 0.277 ± 0.262 | 43.3 ± 55.0 | 17.2 ± 11.8 |
|  | Bioaccessible % | 12.3 ± 7.40 | 20.7 ± 7.03 | 7.13 ± 6.44 | 30.7 ± 18.9 |
| Control | Total | 999 ± 1887 | 1.27 ± 2.00 | 520 ± 828 | 20.6 ± 7.54 |
|  | Bioaccessible | 156 ± 306 | 0.300 ± 0.438 | 63.8 ± 111 | 5.84 ± 0.00 |
|  | Bioaccessible % | 16.6 ± 8.64 | 25.5 ± 8.85 | 8.30 ± 6.54 | 30.7 ± 11.0 |

**Supplementary Table 2: Invertebrate trace metal concentrations across the mine, private property, and control sites.** Means are reported as ± standard deviation, ranges are provided in italics. Concentrations are in mg kg^-1^.

| **Site** | **n** | **Pb** | **Cd** | **Zn** | **Cu** |
| --- | --- | --- | --- | --- | --- |
| Mine | 75 | 1830 ± 2520 | 7.20 ± 6.07 | 2020 ± 1460 | 32.0 ± 18.2 |
|  |  | *79.5 – 15900* | *0.52 – 38.7* | *217 – 6650* | *1.92 – 106* |
| Private Property | 35 | 3050 ± 2980 | 9.52 ± 5.97 | 2380 ± 1890 | 47.3 ± 33.3 |
|  |  | *141 – 10200* | *0.87 – 28.8* | *107 – 8890* | *12.6 – 130* |
| Control | 88 | 52.9 ± 140 | 1.81 ± 1.11 | 543 ± 296 | 32.3 ± 29.4 |
|  |  | *2.24 – 921* | *0.23 – 5.18* | *118 – 1620* | *10.2 – 254* |

**Supplementary Table 3: Wood mouse trace metal concentrations across the mine, private property, and control sites.** Means are reported as ± standard deviation, ranges are provided in italics. Concentrations are in mg kg^-1^ dry weight.

| **Site** | **Tissue** | **n** | **Pb** | **Cd** | **Zn** | **Cu** |
| --- | --- | --- | --- | --- | --- | --- |
| Mine | Kidney | 21 | 42.8 ± 107 | 2.16 ± 2.68 | 107 ± 52.4 | 20.0 ± 10.6 |
|  |  |  | *3.09 – 503* | *0.0925 – 11.4* | *34.0 – 205* | *7.59 – 43.6* |
|  | Liver | 30 | 3.47 ± 4.36 | 0.337 ± 0.510 | 77.2 ± 34.3 | 12.9 ± 4.99 |
|  |  |  | *0.156 – 19.0* | *0.0293 – 2.36* | *24.2 – 140* | *2.94 – 23.0* |
|  | Bone | 28 | 220 ± 265 | 0.0244 ± 0.0369 | 158 ± 32.0 | 1.74 ± 0.737 |
|  |  |  | *6.77 – 1090* | *0.00336 – 0.199* | *91.7 – 223* | *0.680 – 3.16* |
| Private Property | Kidney | 50 | 12.5 ± 19.7 | 2.24 ± 3.96 | 80.8 ± 45.8 | 15.2 ± 7.94 |
|  |  |  | *0.642 – 134* | *0.0914 – 26.3* | *7.28 – 212* | *1.35 – 42.8* |
|  | Liver | 50 | 2.31 ± 2.48 | 0.485 ± 0.606 | 83.8 ± 21.3 | 14.0 ± 3.49 |
|  |  |  | *0.221 – 10.3* | *0.0324 – 3.14* | *33.6 – 161* | *5.16 – 25.8* |
|  | Bone | 50 | 109 ± 198 | 0.0113 ± 0.0124 | 153 ± 51.0 | 1.63 ± 0.970 |
|  |  |  | *3.97 – 1080* | *0.00377 – 0.0686* | *15.4 – 327* | *0.151 – 5.40* |
| Control | Kidney | 27 | 0.887 ± 0.618 | 1.25 ± 2.01 | 98.0 ± 24.0 | 18.6 ± 6.35 |
|  |  |  | *0.211 – 2.50* | *0.0672 – 8.70* | *73.1 – 186* | *12.0 – 45.4* |
|  | Liver | 31 | 0.220 ± 0.270 | 0.259 ± 0.379 | 105 ± 45.5 | 15.8 ± 5.12 |
|  |  |  | *0.0220 – 1.37* | *0.0284 – 1.70* | *22.2 – 213* | *4.28 – 25.7* |
|  | Bone | 30 | 2.92 ± 4.85 | 0.0109 ± 0.0194 | 174 ± 63.4 | 3.05 ± 2.32 |
|  |  |  | *0.191 – 26.2* | *0.00336 – 0.107* | *18.2 – 406* | *0.151 – 11.4* |

**Supplementary Table 4: Significance table of wood mouse tissue trace metal concentrations across factors**. All relationships were calculated using ANOVAs modelling metal concentration across site type (mine, private property, or control), sex (female or male), age class (juvenile or adult), collection time (May 2019, October 2019, September 2021), location (Area 1 or Area 2), and individual site (Mine Complex 1, Mine 2, Private Property 1, Private Property 2, Control 1, Control 2). The reported p-values are adjusted following Yekutieli & Benjamini (1999). A star signifies a significant p-value.

| **Metal** | **Tissue** | **Site Type** | **Collection Month** | **Location** | **Age** | **Sex** | **Individual Site** |
| --- | --- | --- | --- | --- | --- | --- | --- |
| Pb | Kidney | F = 103 | F = 9.48 | F = 23.3 | F = 11.1 | F = 0.352 | F = 0.964 |
|  |  | *p > 0.0001** | *p = 0.00796** | *p > 0.0001** | *p = 0.00438** | *p = 0.624* | *p = 0.455* |
|  | Liver | F = 71.1 | F = 8.47 | F = 20.7 | F = 2.11 | F = 4.293 | F = 1.17 |
|  |  | *p > 0.0001** | *p = 0.00159** | *p =0.000119** | *p = 0.210* | *p = 0.0668* | *p = 0.390* |
|  | Bone | F = 112 | F = 4.59 | F = 14.6 | F = 6.52 | F = 3.80 | F = 1.50 |
|  |  | *p > 0.0001** | *p = 0.0228** | *p = 0.00120** | *p = 0.0238** | *p = 0.140* | *p = 0.309* |
| Cd | Kidney | F = 4.27 | F = 4.43 | F = 13.8 | F = 5.43 | F = 7.19 | F = 4.77 |
|  |  | *p = 0.0320** | *p = 0.0638* | *p = 0.00151** | *p = 0.0387** | *p = 0.0185** | *p = 0.0222** |
|  | Liver | F = 5.13 | F = 4.06 | F = 8.95 | F = 3.93 | F = 7.94 | F = 3.06 |
|  |  | *p = 0.0170** | *p = 0.0371** | *p = 0.00866** | *p =0.0801* | *p = 0.0137** | *p = 0.0805* |
|  | Bone | F = 6.94 | F = 8.89 | F = 0.138 | F = 0.800 | F = 1.37 | F = 11.9 |
|  |  | *p = 0.00484** | *p = 0.00137** | *p = 0.776* | *p = 0.448* | *p = 0.327* | *p = 0.000167** |
| Zn | Kidney | F = 7.60 | F = 30.7 | F = 3.179 | F = 15.9 | F = 0.0480 | F = 1.06 |
|  |  | *p = 0.00324** | *p > 0.0001** | *p = 0.117* | *p = 0.000748** | *p = 0.862* | *p = 0.426* |
|  | Liver | F = 11.0 | F = 78.8 | F = 5.54 | F = 24.3 | F = 3.76 | F = 1.23 |
|  |  | *p = 0.000294** | *p > 0.0001** | *p = 0.0371** | *p > 0.0001** | *p = 0.0846* | *p = 0.374* |
|  | Bone | F = 0.918 | F = 8.21 | F = 0 | F = 0.0390 | F = 0.218 | F = 1.41 |
|  |  | *p = 0.468* | *p = 0.00193** | *p = 0.992* | *p = 0.869* | *p = 0.661* | *p = 0.327* |
| Cu | Kidney | F = 6.28 | F = 19.3 | F = 7.47 | F = 10.5 | F = 1.97 | F = 0.224 |
|  |  | *p = 0.00796** | *p = 0.000200** | *p = 0.0135** | *p = 0.00510** | *p = 0.227* | *p = 0.856* |
|  | Liver | F = 6.05 | F = 34.3 | F = 9.31 | F = 10.7 | F = 0.644 | F = 2.46 |
|  |  | *p = 0.00846** | *p > 0.0001** | *p = 0.00796** | *p = 0.00484** | *p = 0.485* | *p = 0.133* |
|  | Bone | F = 8.86 | F = 3.43 | F = 7.99 | F = 1.33 | F = 0.0190 | F = 0.228 |
|  |  | *p = 0.00137** | *p = 0.0621* | *p = 0.0137** | *p = 0.327* | *p = 0.902* | *p = 0.856* |

**Supplementary Table 5: Wood mouse trace metal concentrations across analysed factors.** Means are reported as ± standard deviation, ranges are provided in italics. Concentrations are in mg kg^-1^ dry weight.

| **Factor** |  | **Tissue** | **n** | **Pb** | **Cd** | **Zn** | **Cu** |
| --- | --- | --- | --- | --- | --- | --- | --- |
| **Collection Month** | May 2019 | Kidney | 37 | 8.01 ± 7.98 | 2.46 ± 4.52 | 60.2 ± 17.8 | 12.1 ± 3.20 |
|  |  |  |  | *0.642 – 41.8* | *0.0914 – 26.3* | *7.28 – 107* | *1.35 – 20.9* |
|  |  | Liver | 37 | 2.12 ± 2.42 | 0.537 ± 0.650 | 74.4 ± 14.9 | 13.0 ± 2.08 |
|  |  |  |  | *0.221 – 10.3* | *0.0324 – 3.14* | *48.3 – 107* | *8.71 – 17.7* |
|  |  | Bone | 37 | 60.6 ± 71.9 | 0.0100 ± 0.0139 | 132 ± 36.8 | 1.45 ± 0.960 |
|  |  |  |  | *3.97 – 298* | *0.00377 – 0.0686* | *15.4 – 208* | *0.151 – 5.40* |
|  | October 2019 | Kidney | 61 | 20.5 ± 66.4 | 1.64 ± 2.18 | 110 ± 44.0 | 20.3 ± 9.00 |
|  |  |  |  | *0.211 – 503* | *0.0672 – 11.4* | *57.4 – 212* | *10.5 – 45.4* |
|  |  | Liver | 60 | 2.34 ± 3.61 | 0.340 ± 0.481 | 108 ± 30.7 | 16.3 ± 4.23 |
|  |  |  |  | *0.0220 – 19.1* | *0.0284 – 2.36* | *33.6 – 213* | *5.16 – 25.8* |
|  |  | Bone | 62 | 136 ± 257 | 0.0172 ± 0.0284 | 175 ± 51.6 | 2.44 ± 1.74 |
|  |  |  |  | *0.191 – 1090* | *0.00336 – 0.199* | *18.2 – 406* | *0.151 – 11.4* |
|  | September 2021 | Kidney | 0 | – | – | – | – |
|  |  |  |  | – | – | – | – |
|  |  | Liver | 14 | 0.530 ± 0.743 | 0.147 ± 0.156 | 38.8 ± 17.0 | 8.34 ± 4.05 |
|  |  |  |  | *0.0319 – 2.48* | *0.0293 – 0.582* | *22.2 – 86.4* | *2.94 – 15.8* |
|  |  | Bone | 8 | 125 ± 141 | 0.0166 ± 0.0131 | 170 ± 54.5 | 1.68 ± 1.23 |
|  |  |  |  | *1.51 – 451* | *0.00377 – 0.0429* | *116 – 296* | *0.680 – 4.43* |

| **Factor** |  | **Tissue** | **n** | **Pb** | **Cd** | **Zn** | **Cu** |
| --- | --- | --- | --- | --- | --- | --- | --- |
| **Location** | Area 1 | Kidney | 41 | 5.61 ± 5.26 | 1.26 ± 2.03 | 78.3 ± 33.9 | 13.6 ± 3.15 |
|  |  |  |  | *0.211 – 22.5* | *0.0672 – 11.4* | *34.0 – 212* | *7.59 – 20.9* |
|  |  | Liver | 45 | 1.27 ± 1.71 | 0.279 ± 0.415 | 81.9 ± 33.3 | 13.0 ± 4.16 |
|  |  |  |  | *0.0319 – 7.94* | *0.0284 – 2.36* | *28.4 – 213* | *4.00 – 24.9* |
|  |  | Bone | 44 | 50.6 ± 69.4 | 0.0167 ± 0.0306 | 161 ± 64.7 | 1.78 ± 1.90 |
|  |  |  |  | *0.191 – 332* | *0.00377 – 0.199* | *18.2 – 406* | *0.151 – 11.4* |
|  | Area 2 | Kidney | 57 | 23.1 ± 68.4 | 2.45 ± 3.86 | 100 ± 47.7 | 19.8 ± 9.91 |
|  |  |  |  | *0.254 – 503* | *0.154 – 26.3* | *7.28 – 209* | *1.35 – 45.4* |
|  |  | Liver | 66 | 2.56 ± 3.61 | 0.452 ± 0.589 | 92.2 ± 35.2 | 15.1 ± 4.57 |
|  |  |  |  | *0.0220 – 19.1* | *0.0337 – 3.14* | *22.2 – 192* | *2.94 – 25.8* |
|  |  | Bone | 63 | 150 ± 255 | 0.0132 ± 0.0170 | 159 ± 39.3 | 2.23 ± 1.22 |
|  |  |  |  | *0.596 – 1090* | *0.0336 – 0.107* | *15.4 – 296* | *0.151 – 9.06* |
| **Age** | Juvenile | Kidney | 42 | 26.3 ± 79.4 | *1.27* ± 2.02 | *113* ± 49.8 | *21.1* ± 10.2 |
|  |  |  |  | *0.211 – 503* | *0.0672 – 11.4* | *34.0 – 212* | *7.59 – 45.4* |
|  |  | Liver | 47 | 2.41 ± 3.86 | 0.261 ± 0.426 | 96.8 ± 34.1 | 15.2 ± 4.59 |
|  |  |  |  | *0.0220 – 19.1* | *0.0284 – 2.36* | *29.1 – 192* | *4.00 – 25.7* |
|  |  | Bone | 46 | 154 ± 270 | 0.0152 ± 0.0296 | 164 ± 41.4 | 2.19 ± 1.02 |
|  |  |  |  | *0.191 – 1090* | *0.00336 – 0.199* | *18.2 – 327* | *0.151 – 6.65* |

| **Factor** |  | **Tissue** | **n** | **Pb** | **Cd** | **Zn** | **Cu** |
| --- | --- | --- | --- | --- | --- | --- | --- |
| **Age** | Adult | Kidney | 56 | 7.91 ± 7.95 | 2.46 ± 3.89 | 74.6 ± 29.4 | 14.2 ± 5.04 |
|  |  |  |  | *0.226 – 41.8* | *0.0914 – 26.3* | *7.28 – 209* | *1.35 – 39.3* |
|  |  | Liver | 64 | 1.76 ± 2.27 | 0.470 ± 0.582 | 81.6 ± 33.8 | 13.5 ± 4.33 |
|  |  |  |  | *0.0319 – 10.3* | *0.0324 – 3.14* | *22.2 – 213* | *2.94 – 25.8* |
|  |  | Bone | 61 | 75.9 ± 132 | 0.0143 ± 0.0178 | 157 ± 57.3 | 1.93 ± 1.84 |
|  |  |  |  | *0.416 – 829* | *0.00336 – 0.107* | *15.4 – 406* | *0.151 – 11.4* |
| **Sex** | Female | Kidney | 40 | 23.9 ± 79.3 | 2.78 ± 4.55 | 92.3 ± 46.7 | 16.3 ± 8.18 |
|  |  |  |  | *0.227 – 503* | *0.151 – 26.3* | *7.28 – 212* | *1.35 – 43.6* |
|  |  | Liver | 45 | 2.79 ± 3.92 | 0.507 ± 0.638 | 92.9 ± 39.4 | 14.8 ± 5.54 |
|  |  |  |  | *0.0220 – 19.1* | *0.0293 – 3.14* | *24.2 – 213* | *2.94 – 25.8* |
|  |  | Bone | 43 | 156 ± 258 | 0.0187 ± 0.0338 | 157 ± 54.1 | 2.12 ± 1.96 |
|  |  |  |  | *0.416 - 1090* | *0.00336 – 0.199* | *15.4 – 406* | *0.151 – 11.4* |
|  | Male | Kidney | 58 | 10.2 ± 18.9 | 1.38 ± 1.79 | 90.5 ± 41.8 | 17.8 ± 8.51 |
|  |  |  |  | *0.211 – 135* | *0.0672 – 7.70* | *34.0 – 207* | *7.59 – 43.4* |
|  |  | Liver | 66 | 1.53 ± 2.16 | 0.296 ± 0.426 | 84.6 ± 30.8 | 13.8 ± 3.62 |
|  |  |  |  | *0.0220 – 10.3* | *0.0284 – 2.29* | *22.2 – 192* | *4.28 – 25.7* |
|  |  | Bone | 64 | 78.3 ± 157 | 0.0119 ± 0.0121 | 162 ± 49.1 | 1.99 ± 1.21 |
|  |  |  |  | *0.191 - 1080* | *0.00336 – 0.0448* | *18.2 – 327* | *0.151 – 6.65* |

| **Factor** |  | **Tissue** | **n** | **Pb** | **Cd** | **Zn** | **Cu** |
| --- | --- | --- | --- | --- | --- | --- | --- |
| **Individual Sites** | Mine Complex 1 | Kidney | 8 | 7.88 ± 4.83 | 2.46 ± 3.86 | 75.8 ± 33.5 | 13.5 ± 4.67 |
|  |  |  |  | *3.09 – 15.3* | *0.0925 – 11.4* | *34.0 – 117* | *7.59 – 20.9* |
|  |  | Liver | 12 | 1.49 ± 1.99 | 0.347 ± 0.647 | 58.9 ± 25.0 | 9.76 ± 3.73 |
|  |  |  |  | *0.156 – 6.58* | *0.0293 – 2.36* | *28.4 – 97.8* | *4.00 – 13.8* |
|  |  | Bone | 11 | 104 ± 109 | 0.0408 ± 0.0547 | 139 ± 34.7 | 1.28 ± 0.796 |
|  |  |  |  | *6.77 – 332* | *0.00377 – 0.195* | *91.7 – 223* | *0.680 – 3.16* |
|  |  | Kidney | 25 | 6.49 ± 5.32 | 0.838 ± 1.14 | 72.3 ± 35.4 | 13.2 ± 2.55 |
|  |  |  |  | *0.759 – 22.7* | *0.0914 – 5.79* | *43.6 – 212* | *9.17 – 19.9* |
|  | Private Property 1 | Liver | 25 | 1.50 ± 1.76 | 0.246 ± 0.332 | 84.0 ± 24.9 | 13.7 ± 3.40 |
|  |  |  |  | *0.221 – 7.94* | *0.0324 – 1.63* | *33.6 – 161* | *5.16 – 24.9* |
|  |  | Bone | 25 | 42.6 ± 39.8 | 0.00721 ± 0.00695 | 162 ± 55.1 | 1.41 ± 1.01 |
|  |  |  |  | *4.68 – 143* | *0.00377 – 0.0282* | *101 – 327* | *0.412 – 5.40* |
|  | Control 1 | Kidney | 8 | 0.587 ± 0.336 | 1.39 ± 1.38 | 99.8 ± 22.1 | 15.0 ± 3.14 |
|  |  |  |  | *0.211 – 1.08* | *0.0672 – 3.67* | *73.1 – 130* | *12.0 – 20.8* |
|  |  | Liver | 8 | 0.201 ± 0.182 | 0.282 ± 0.191 | 110 ± 45.2 | 15.7 ± 4.42 |
|  |  |  |  | *0.0319 – 0.485* | *0.0284 – 0.540* | *75.0 – 213* | *11.7 – 23.7* |
|  |  | Bone | 8 | 2.70 ± 3.04 | 0.0134 ± 0.00768 | 189 ± 109 | 3.63 ± 3.62 |
|  |  |  |  | *0.191 – 8.30* | *0.00377 – 0.0250* | *18.2 – 406* | *0.151 – 11.2* |

| **Factor** |  | **Tissue** | **n** | **Pb** | **Cd** | **Zn** | **Cu** |
| --- | --- | --- | --- | --- | --- | --- | --- |
| **Individual Sites** | Mine 2 | Kidney | 13 | 64.3 ± 133 | 1.97 ± 1.77 | 126 ± 53.6 | 24.1 ± 11.3 |
|  |  |  |  | *5.85 – 503* | *0.319 – 6.06* | *77.7 – 205* | *14.5 – 43.6* |
|  |  | Liver | 18 | 4.78 ± 5.03 | 0.330 ± 0.416 | 89.3 ± 34.8 | 15.0 ± 4.65 |
|  |  |  |  | *0.244 – 19.1* | *0.0373 – 1.75* | *24.2 – 140* | *2.94 – 23.0* |
|  |  | Bone | 17 | 296 ± 309 | 0.0138 ± 0.0110 | 171 ± 23.5 | 2.04 ± 0.625 |
|  |  |  |  | *17.1 – 1090* | *0.00336 – 0.0447* | *139 – 217* | *1.27 – 2.87* |
|  | Private Property 2 | Kidney | 25 | 18.5 ± 26.3 | 3.65 ± 5.16 | 89.4 ± 53.7 | 17.2 ± 10.7 |
|  |  |  |  | *0.641 – 135* | *0.276 – 26.3* | *7.28 – 209* | *1.35 – 42.8* |
|  |  | Liver | 25 | 3.12 ± 2.84 | 0.724 ± 0.721 | 83.6 ± 17.3 | 14.4 ± 3.61 |
|  |  |  |  | *0.737 – 10.3* | *0.0690 – 3.14* | *55.8 – 126* | *3.61 – 8.90* |
|  |  | Bone | 25 | 175 ± 263 | 0.0155 ± 0.0152 | 144 ± 45.8 | 1.85 ± 0.898 |
|  |  |  |  | *3.97 – 1080* | *0.00377 – 0.0686* | *15.4 – 249* | *0.151 – 4.88* |
|  | Control 2 | Kidney | 19 | 1.01 ± 0.671 | 1.19 ± 2.25 | 97.2 ± 25.3 | 20.1 ± 6.80 |
|  |  |  |  | *0.254 – 2.50* | *0.154 – 8.70* | *77.5 – 186* | *13.7 – 45.4* |
|  |  | Liver | 23 | 0.227 ± 0.298 | 0.251 ± 0.430 | 104 ± 46.5 | 15.8 ± 5.43 |
|  |  |  |  | *0.0220 – 1.37* | *0.0337 – 1.70* | *22.2 – 192* | *4.28 – 25.7* |
|  |  | Bone | 21 | 3.00 ± 5.44 | 0.0100 ± 0.0224 | 168 ± 36.6 | 2.84 ± 1.67 |
|  |  |  |  | *0.596 – 26.2* | *0.00336 – 0.107* | *130 – 296* | *1.67 – 1.44* |

### Appendix C: Unified BARGE Method Protocol

Protocol modified from BARGE & INERIS (2010).

**A) Digestive Fluids Preparation**

1. Label eight 500 mL volumetric flasks, two for each digestive fluid (Saliva, Gastric, Duodenal, or Bile).
2. For each digestive fluid, label one flask with “inorganic” and the other flask with “organic”.
3. Add the necessary components to the corresponding bottles, as shown in Supplementary Table 6.
4. Mix each corresponding organic and inorganic solution together in 1 L bottles.
5. Store at room temperature until the day before analysis.
6. Label new bottles for each digestive fluid.
7. Add enzymes to each bottle as specified in Supplementary Table 6, followed by the indicated amount of each digestive fluid.
8. Mix the bottles on spinner plates for 1 hour.
9. Adjust the pH of the digestive fluids using 1M NaOH and 1M HCl solutions until they reach the following pHs:
   1. Saliva: 6.5 ± 0.5
   2. Gastric: 1.1 ± 0.1
   3. Duodenal: 7.4 ± 0.2
   4. Bile: 8 ± 0.2
10. Store overnight at 4˚C.

**B) Unified BARGE Method Test**

1. Weigh out 0.47 g of soil and place in a 50 mL polycarbonate tube.
   1. Run each soil sample in triplicate to account for potential pH variations during the UBM.
2. Add 7.0 mL of Saliva fluid by pipette.
3. Shake by hand for approximately 10 seconds.
4. Add 10.5 mL of Gastric fluid by pipette.
5. Check pH = 1.20 ± 0.05. Adjust with NaOH 1M and/or HCl 37% if necessary.
6. Shake by hand for approximately 10 seconds.
7. Check pH = 1.20 ± 0.05. Adjust with NaOH 1M and/or HCl 37% if necessary.
8. Place tubes into an incubator set to 37˚C and 200 shakes per minute.
9. Leave the samples in the incubator for 1 hour.
10. Check if pH < 1.50 for each sample. If pH < 1.50, note the pH.
11. Add 21 mL of Duodenal fluid by pipette.
12. Add 7.0 mL of Bile fluid by pipette.
13. Check pH = 6.30 ± 0.5. Adjust with NaOH 1M and/or HCl 37% if necessary.
14. Place tubes into an incubator set to 37˚C and 200 shakes per minute.
15. Leave the samples in the incubator for 4 hours.
16. Note the final pH of the extracts.
17. Centrifuge the samples for 15 minutes at 4500 g.
18. Collect the top 20 mL of the supernatant.
19. Store supernatant at -20˚C until ICP-MS analysis.

**Supplementary Table 6: Digestive fluid reagents for the UBM.** Numbers in italics are in mL, while numbers not in italics are in g.

| **Reagent** |  | **Saliva** | **Gastric** | **Duodenal** | **Bile** |
| --- | --- | --- | --- | --- | --- |
| **INORGANIC** | |  |  |  |  |
| KCl | Potassium chloride | 0.896 | 0.824 | 0.564 | 0.376 |
| NaH_2_PO_4_ | Sodium dihydrogen phosphate dihydrate | 0.888 | 0.266 |  |  |
| KSCN | Potassium thiocynate | 0.200 |  |  |  |
| Na_2_SO_4_ | Sodium sulfate | 0.570 |  |  |  |
| NaCl | Sodium chloride | 0.298 | 2.752 | 7.012 | 5.26 |
| CaCl_2_ | Calcium chloride dehydrate |  | 400 |  |  |
| NH_4_Cl | Ammonium chloride |  | 306 |  |  |
| NaHCO_3_ | Sodium hydrogen carbonate |  |  | 5.607 | 5.786 |
| KH_2_PO_4_ | Potassium dihydrogen orthophoshate |  |  | 0.080 |  |
| MgCl_2_ | Magnesium chloride |  |  | 0.050 |  |
| 1 M NaOH | Sodium hydroxide | *1.8* |  |  |  |
| 37% HCl | Hydrochloric acid |  | *8.3* | *0.180* | *0.180* |
| Pure H_2_O | Milli-Q Water | *500* | *500* | *500* | *500* |
| **ORGANIC** | |  |  |  |  |
|  | Urea | 0.200 | 0.085 | 0.100 | 0.250 |
|  | Glucose |  | 0.650 |  |  |
|  | Glucuronic acid |  | 0.020 |  |  |
|  | Glucosamine hydrochloride |  | 0.330 |  |  |
| Pure H_2_O | Milli-Q Water | *500* | *500* | *500* | *500* |

**Supplementary Table 6: Digestive fluid enzymes for the UBM.** Numbers in italics are in mL, while numbers not in italics are in g.

| **Enzyme** | **Saliva** | **Gastric** | **Duodenal** | **Bile** |
| --- | --- | --- | --- | --- |
| Alpha amylase | 0.029 |  |  |  |
| Mucin | 0.010 | 0.900 |  |  |
| Uric acid | 0.003 |  |  |  |
| Bovine Serum Albumin |  | 0.300 | 0.600 | 0.360 |
| Pepsin |  | 0.300 |  |  |
| CaCl_2_ |  |  | 0.120 | 0.044 |
| Pancreatin |  |  | 1.800 |  |
| Lipase |  |  | 0.300 |  |
| Bile |  |  |  | 1.200 |
| Digestive Fluid | *200* | *300* | *600* | *200* |
